# Ternary Cu_2_SnS_3_: Synthesis, Structure, Photoelectrochemical Activity, and Heterojunction Band Offset and Alignment

**DOI:** 10.1021/acs.chemmater.0c03223

**Published:** 2021-03-03

**Authors:** Sagar B. Jathar, Sachin R. Rondiya, Yogesh A. Jadhav, Dhanaraj S. Nilegave, Russell W. Cross, Sunil V. Barma, Mamta P. Nasane, Shankar A. Gaware, Bharat R. Bade, Sandesh R. Jadkar, Adinath M. Funde, Nelson Y. Dzade

**Affiliations:** † School of Energy Studies, Savitribai Phule Pune University, Pune 411007, India; § School of Chemistry, Cardiff University, Main Building, Park Place, Cardiff, CF10 3AT, Wales, United Kingdom; ‡ Department of Physics, Savitribai Phule Pune University, Pune 411007, India

## Abstract

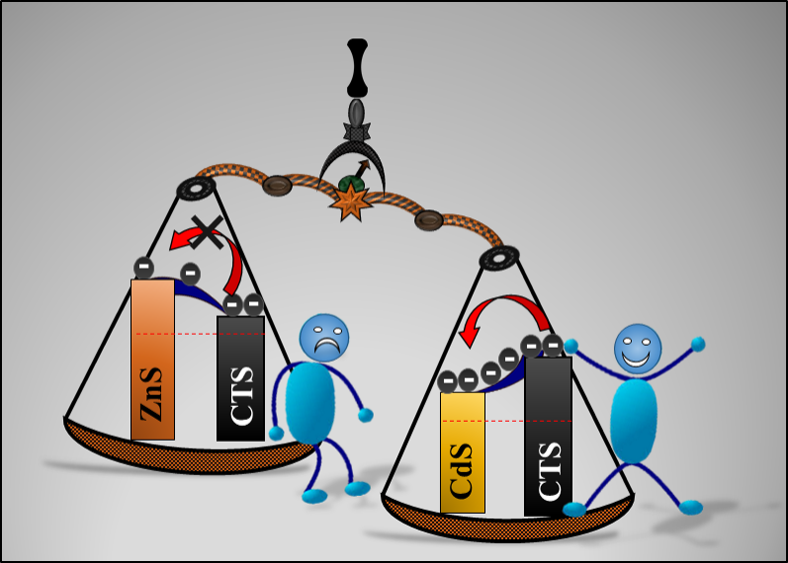

Ternary Cu_2_SnS_3_ (CTS) is an attractive nontoxic and earth-abundant absorber material with suitable optoelectronic properties for cost-effective photoelectrochemical applications. Herein, we report the synthesis of high-quality CTS nanoparticles (NPs) using a low-cost facile hot injection route, which is a very simple and nontoxic synthesis method. The structural, morphological, optoelectronic, and photoelectrochemical (PEC) properties and heterojunction band alignment of the as-synthesized CTS NPs have been systematically characterized using various state-of-the-art experimental techniques and atomistic first-principles density functional theory (DFT) calculations. The phase-pure CTS NPs confirmed by X-ray diffraction (XRD) and Raman spectroscopy analyses have an optical band gap of 1.1 eV and exhibit a random distribution of uniform spherical particles with size of approximately 15–25 nm as determined from high-resolution transmission electron microscopy (HR-TEM) images. The CTS photocathode exhibits excellent photoelectrochemical properties with PCE of 0.55% (fill factor (FF) = 0.26 and open circuit voltage (Voc) = 0.54 V) and photocurrent density of −3.95 mA/cm^2^ under AM 1.5 illumination (100 mW/cm^2^). Additionally, the PEC activities of CdS and ZnS NPs are investigated as possible photoanodes to create a heterojunction with CTS to enhance the PEC activity. CdS is demonstrated to exhibit a higher current density than ZnS, indicating that it is a better photoanode material to form a heterojunction with CTS. Consistently, we predict a staggered type-II band alignment at the CTS/CdS interface with a small conduction band offset (CBO) of 0.08 eV compared to a straddling type-I band alignment at the CTS/ZnS interface with a CBO of 0.29 eV. The observed small CBO at the type-II band aligned CTS/CdS interface points to efficient charge carrier separation and transport across the interface, which are necessary to achieve enhanced PEC activity. The facile CTS synthesis, PEC measurements, and heterojunction band alignment results provide a promising approach for fabricating next-generation Cu-based light-absorbing materials for efficient photoelectrochemical applications.

## 1 Introduction

Scaling up of renewable energy generation is crucial for the decarbonization of the world’s energy systems. Photovoltaic solar energy technology has become a major electricity generation source, and it is expected to lead the way in the transformation of the global electricity sector.^1,2^ The ever-growing demands of cost-effective and high-efficiency solar cells have prompted an unceasing search for nontoxic, earth-abundant, and stable solar absorber materials. Chalcogenide thin films such as CIGS (Cu_2_InGaSe_4_) and CdTe have been intensively investigated, demonstrating a photovoltaic cell efficiency of η = 22.6 and 22.1%, respectively.^3,4^ However, the scarcity, cost, and toxicity associated with the In, Ga, and Cd elements present in these cells limit their sustainable future applications.^5,6^ A quaternary system, Cu_2_ZnSn(S/Se)_4_,^7^ with superior optoelectronic properties, strong stability, suitable direct band gap (E_g_ = 1.16–1.5 eV), high absorption coefficient (10^4^ cm^–1^), and efficient charge transport and high mobility, has also attracted much attention in the fields of photocurrent conversion.^8−12^ However, the difficulty associated with controlling the inherent Cu–Zn anti-site defects in CZTS(Se) and the formation of secondary phases remain as major limitations to achieving high-performance CZTS/Se photovoltaic and photoelectrochemical devices.^13−15^

Ternary Cu_2_SnS_3_ (CTS), which provides an alternative to rare metal elements and avoids complex preparation processes, is a promising substitute for the quaternary systems.^16,17^ CTS is a p-type semiconductor with a direct band gap (0.93–1.35 eV) and high absorption coefficient (10^4^ cm^–1^), making it a suitable absorber material for PEC applications. Furthermore, the constituent elements of CTS are earth-abundant and nontoxic, making it an attractive environmentally friendly and cost-effective photoabsorber for practical applications.^18^ Among the Cu–Sn–S family, CTS has been identified as the most suitable compound because of its wide stability range and lack of Fermi level pinning.^19^ The photovoltaic performance of CTS thin films fabricated by direct evaporation method was first characterized by Titilayo *et al.* who reported a 0.11% device efficiency.^20^ Recently, Nakashima *et al*. reported a 4.67% conversion efficiency for a CTS solar cell by the vacuum evaporation method, and Mitsutaro *et al*. achieved 6% for a Ge doped CTS solar cell.^21,22^ These efficiencies are, however, significantly lower than the reported theoretical efficiency of 30% by Avellaneda *et al*.^23^ Therefore, it is quite clear that there is much scope and further studies are needed to improve the performance of CTS photovoltaic and photoelectrochemical (PEC) devices. There exist only a few reports on the PEC measurement of CTS in the literature. Shelke *et al.* performed PEC measurement on CTS films and observed an efficiency of 0.11% for the short circuit photocurrent density (Jsc) of 0.46 mA cm^–2^ and fill factor (FF) of 30.1.^24^ In another study, Shelke *et al.* studied the photoelectric properties of CTS thin films of different thicknesses prepared by the SILAR method.^25^ Annealed CTS films have been demonstrated to yield better PEC results with higher fill factor as compared to the as-deposited films on a stainless steel substrate by the chemical bath deposition (CBD) method.^26^ Although CTS is the most studied phase in the ternary Cu–Sn–S system, there is still a lack of detailed knowledge/understanding about band alignment and interface properties, which are critical to making further progress in device development.

In the present study, we present high-quality CTS NPs prepared by a cost-effective and robust hot injection (HI) method. The crystal structure, size distribution, surface morphology, and composition of the as-prepared CTS NPs were characterized using X-ray diffraction (XRD) pattern, Raman spectrum, high-resolution transmission electron microscopy (HR-TEM), X-ray photoelectron spectroscopy (XPS), and other complementary experimental techniques. The photoelectrochemical performance of the CTS films was measured and compared with that of CdS and ZnS photoanode materials. Photoelectrochemical impedance spectroscopy (PEIS) measurements were performed to gain insights into the charge carrier kinetics and transfer processes, and the generated data were used to analyze the lifetime of charge carriers, charge transfer resistance. Through Mott–Schottky analysis, the donor concentration of CTS and the photoanode materials was determined. The current–voltage (*J*–*V*) characteristics were used to evaluate efficiencies of PEC cells. Furthermore, we have systematically studied band offset and alignment at CTS/CdS and CTS/ZnS heterojunctions using cyclic voltammetric measurements corroborated by first-principles DFT calculations, providing the basis for heterojunction engineering to enhance charge carrier transport in CTS-based devices.

## 2 Experimental Section

### 2.1 Chemicals

Copper chloride (CuCl_2_·2H_2_O, >99.99%), tin chloride (SnCl_2_·2H_2_O, >99.99%), sulfur powder (S, >99.99%), cadmium sulfate (CdSO_4_, >99.99%), zinc sulfate (ZnSO_4_·7H_2_O, >99.99%), octadecane (ODE, technical grade, 70%), oleylamine (OLA, technical grade, 70%), isopropyl alcohol (IPA, technical grade, 70%), toluene (technical grade, 70%), dichloromethane (DCM, TLC high-purity grade, ≥99.8%), and tetrabutylammonium perchlorate (TBAP, ≥99.0%) were purchased from Sigma Aldrich. All chemicals were used as received from suppliers without any further purification.

### 2.2 Material Synthesis

**Scheme 1:**
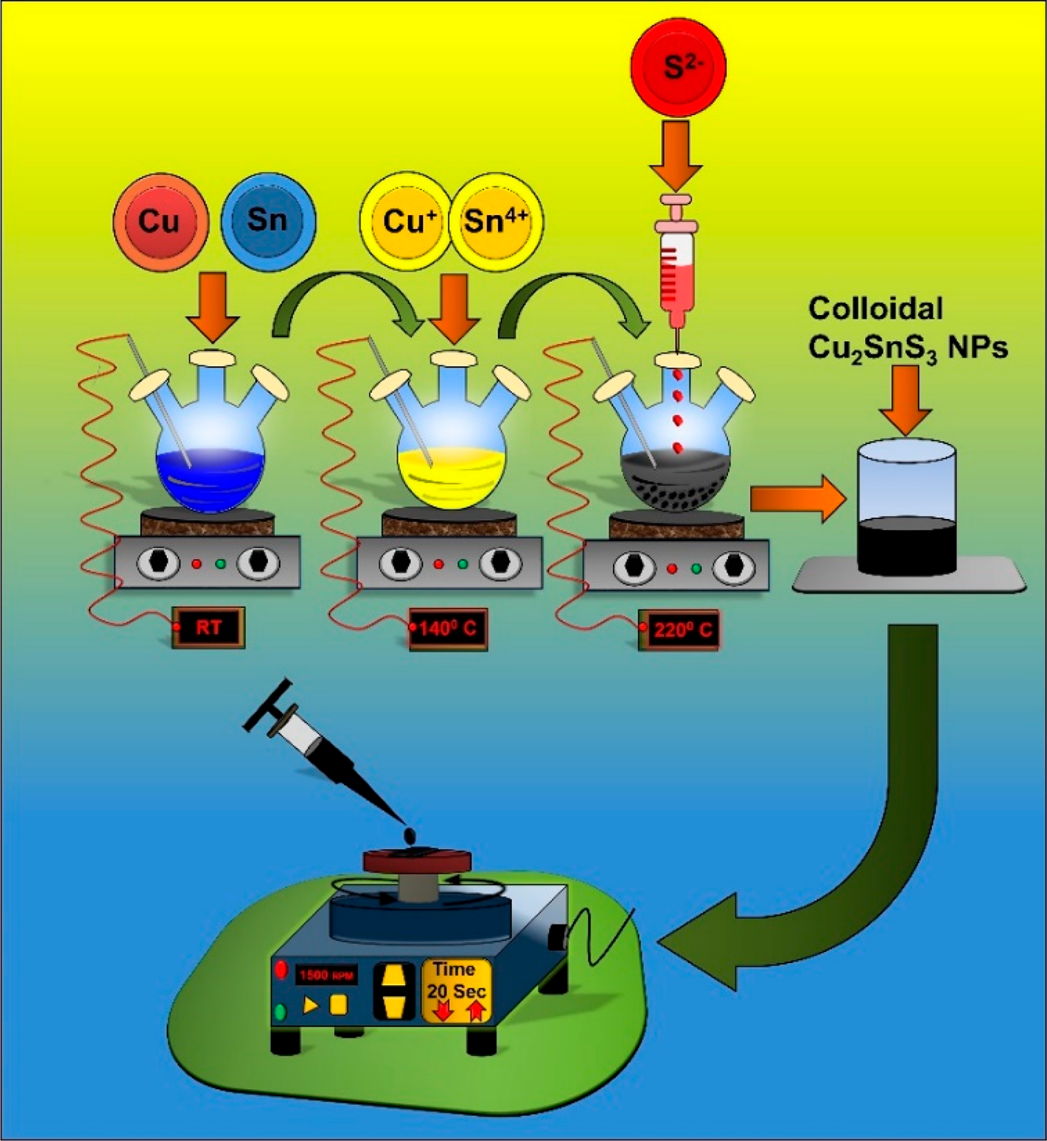
Hot Injection (HI) Synthesis Setup and Different Steps for CTS NPs Synthesis

CTS NPs were prepared via the hot injection (HI) method using ODE as a coordinating solvent. In a typical synthesis, CuCl_2_ (3 mmol) and SnCl_2_ (1.8 mmol) were dissolved in an ODE solution (10 mL) in a three-neck flask with constant stirring at 600 rpm. The solution was allowed to degas under vacuum for 10 minutes. The mixture was then purged with argon for 10 min, and the precursor solution was heated at 140 °C and aged for 20 min, leading to the formation of a yellow Cu–Sn complex. The temperature was raised to 220 °C; simultaneously, sulfur powder (4.5 mmol) was dissolved in 5 mL of octadecane solution at 90 °C. The sulfur solution was swiftly injected into the metal precursor solution at 220 °C, and the reaction was aged for 15 min to enable the growth of CTS NPs. The reaction was then stopped and allowed to cool at room temperature. The reaction mixture was washed with 40 mL of isopropyl alcohol (IPA) and 5 mL of toluene four times for 10 min to remove byproducts. Finally, the obtained CTS powder was dried under IR lamp for 2 h and used for further studies. Details of the hot injection setup and reaction mechanism for the formation of the CTS NPs are shown in Scheme 1. The same synthesis protocol was used for the synthesis of CdS and ZnS NPs. In this case, however, the precursor ratio of 1:1 mmol metal to sulfur was used, and the injection and growth temperature was set to 240 °C. The synthesized NPs were washed, purified, and dried similar to the procedure used for CTS NPs.

### 2.3 Thin-Film Preparation by Spin Coating Technique

The obtained CTS NPs from the HI method were dissolved in a 100 mg/mL toluene solution, and the mixture was sonicated for 3 h to form a thick solution. The resulting solution was used to spin coat 1 × 1 cm fluorine-doped tin oxide (FTO) substrates at 1500 rpm for 20 s. Prior to coating, the substrates were cleaned in a soap solution, distilled water, ethanol, and then acetone followed by air-drying. The deposited films were dried at 150 °C on a hot plate and, after 1 h, were cooled down naturally to room temperature. The same procedure was used to prepare CdS and ZnS films on FTO substrates. The photoelectrochemical properties of the prepared films were systematically investigated.

### 2.4 Material Characterization

The synthesized CTS, CdS, and ZnS NPs were systematically studied using different characterization techniques like X-ray diffraction (XRD), Raman spectroscopy, UV–visible spectroscopy, energy-dispersive X-ray spectroscopy (EDS), and scanning electron microscopy (SEM). The structural properties of the prepared materials were studied using X-ray diffraction (Bruker D8 Advance, Germany) with Cu Kα radiation (λ = 1.54 Å). Raman spectra were recorded using Raman Spectrometer (Renishaw Microscope) with an excitation source of 532.8 nm line of the laser. The optical properties of the prepared samples were estimated from absorbance spectra measured using a JASCO V-670 UV–visible spectrophotometer. The surface morphology of the as-synthesized CTS NPs was investigated using scanning electron microscopy (SEM) performed using a JEOL JSM-6360-LA instrument. The Fourier transform infrared (FTIR) spectra were recorded in the transmission mode by using an FTIR spectrophotometer (Jasco, 6100-type A). The HR-TEM micrographs and selected area electron diffraction (SAED) patterns were obtained with a JEOL-JEM 2100 microscope operating at 200 kV. X-ray photoelectron spectroscopy (XPS) was carried out on the samples using a Kratos Axis Ultra DLD photoelectron spectrometer utilizing monochromatic Al Kα radiation operating at an energy of 120 W (10 × 12 kV). The electrochemical measurements, Mott–Schottky plot (M–S plot), photoelectrochemical impedance spectroscopy (Nyquist and Bode phase plot), cyclic voltammetry (CV), and linear sweep voltammetry (LSV) were carried out using an electrochemical workstation (Metrohm Potentiostat/Galvanostat, Autolab PGSTAT 302N) as per our earlier report.^27−30^ The CTS-based PEC cell was constructed with an FTO/CTS/Na_2_SO_4_/Pt device architecture, and the *J*–*V* characteristics were measured under dark and illuminated conditions. The standard three-electrode system is used, composed of a saturated calomel electrode (SCE), platinum plate, and photoelectrode, which were used as a reference electrode, counter electrode, and working electrode, respectively. The pre-dried 0.341 g of TBAP typically 100 mM in 10 mL of pre-dried DCM was transferred to the electrochemical cell through silicone septa. The blank cyclic voltammograms were recorded for reference in the TBAP–DCM mixture to ensure that there were no peaks corresponding to contamination on the GC electrode. Subsequently, the GC electrode was loaded with 100 μL of sample dispersion in DCM (1.0 mg/mL) and followed by vacuum drying. The scan rate was kept constant (100 mV/s) for all measurements. After completion of each set of experiments, the potentials were calibrated using ferrocene as an internal standard with respect to the normal hydrogen electrode (NHE).^31^

### 2.5 Computational Details

The first-principles density functional theory (DFT) calculations were performed using VASP (Vienna Ab initio Simulation Package).^32−34^ The interactions between the valence electrons and atomic cores were described with the projected augmented wave (PAW) method.^35^ The PBE functional^36^ was used for geometry optimizations, while for electronic structure calculations, the screened hybrid functional HSE06^37^ was used. To accurately reproduce the experimental band gaps and DOS features of CTS, CdS, and ZnS, the exact exchange values of 35, 25, and 30% were used, respectively, with a screening parameter of μ = 0.2 Å^–1^. The projected density of states (PDOS) was calculated using the tetrahedron method with Bloch correction.^38^ An energy cutoff of 600 eV and Monkhorst–Pack^39^*k*-point mesh of 3 × 3 × 3, 7 × 7 × 5, and 7 × 7 × 7 were used to sample the Brillouin zone of the bulk CTS, CdS, and ZnS, respectively. In constructing the cubic CTS structure, a 3 × 3 × 3 supercell of the cubic zinc blende structure was employed to overcome the partial occupancy of Cu and Sn metals, which were distributed at the 4a sites considering three different model arrangements **(**Figure S1, Supplementary Information) in which the Cu and Sn atoms occupy two-thirds and one-third of the 4a sites, respectively.^40−42^ This gives the optimum stoichiometry of Cu_2_SnS_3_. In Model 1, the Sn atoms were distributed over regular two rows separated by Cu atoms, whereas in Model 2, the Sn atoms were distributed over different rows and with less ordering (Figure S1a,b). In Model 3, the Sn atoms were distributed such that they form regular layers separated by Cu atoms (Figure S1c). Energy minimization shows that Model 1 is energetically far more favorable than Model 2 by 4.54 eV and Model 3 by 11.98 eV. Models 1 and 2 exhibit semiconducting characteristics with a predicted band gap of 1.07 and 0.78 eV, respectively (Figure S2a,b), whereas Model 3 shows a metallic behavior (Figure S2c). ZnS was modeled in the cubic zinc blende phase (Figure S3a) and CdS in the hexagonal wurtzite phase (Figure S4a).

To align the energies to the vacuum level, a slab-gap model was constructed and the corresponding electrostatic potential (Figures S5–S7) was averaged along the c-direction using the MacroDensity package.^43−45^ The CTS(111), CdS(100), and ZnS(110) surfaces were chosen for the slab calculations as they do not contain dangling bonds and resulted in low-energy, nonpolar terminations. The slabs were constructed with a thickness > 15 Å, and in every simulation cell, a vacuum region of 20 Å perpendicular to the surface was tested to be sufficient to avoid interactions between periodic slabs. The ionization potentials (IPs) were calculated when the slab vacuum level is aligned to the bulk eigenvalues, through core-level eigenvalues in the center of the slab, using the S 1s orbital energy as a reference point. The electron affinity (EA) is calculated by subtracting the band gaps from the calculated IPs.

## 3 Results and Discussion

### 3.1 Structural and Optical Properties of CTS NPs

**Figure 1:**
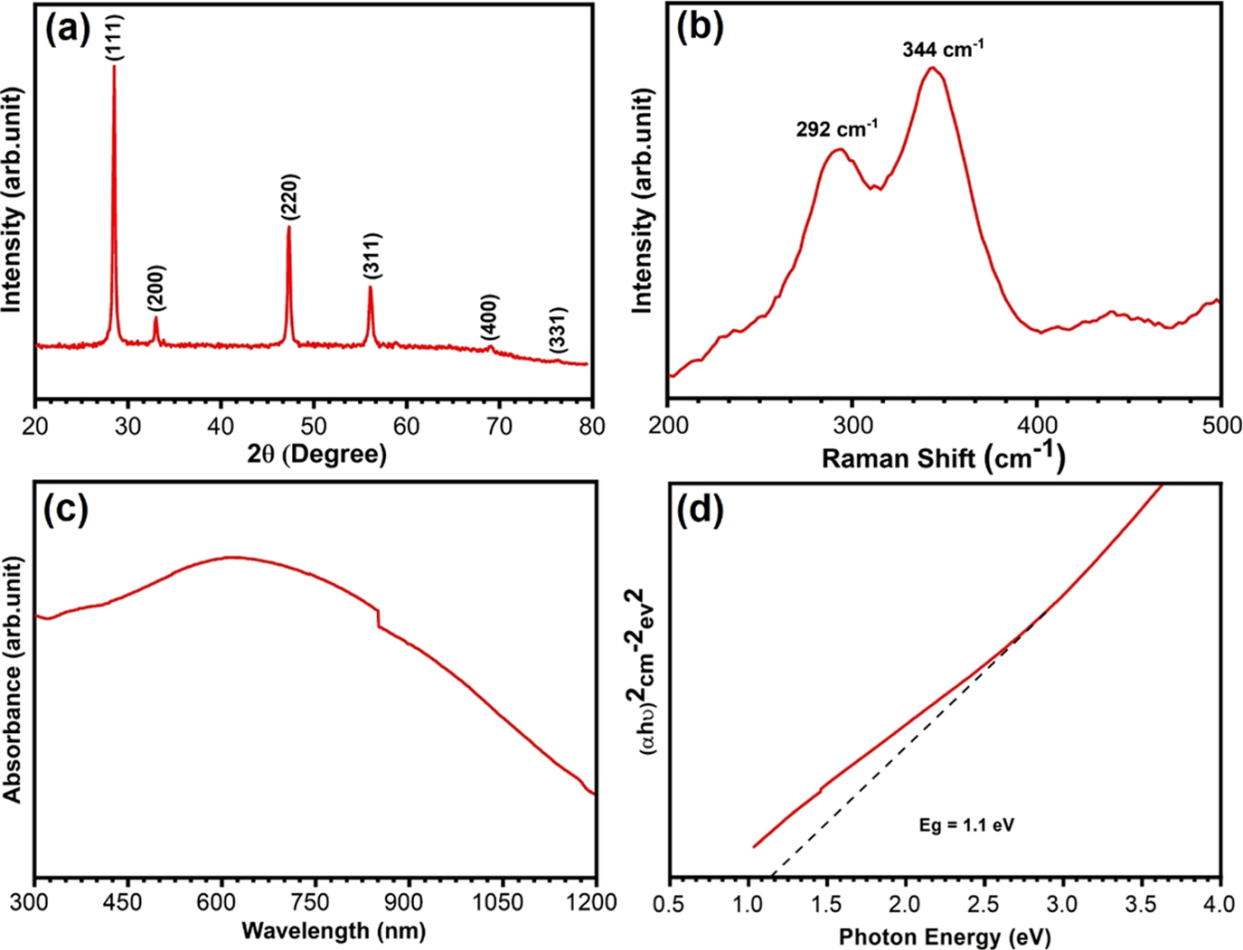
(a) XRD pattern of CTS NPs synthesized by facile hot injection method. (b) Raman spectrum of CTS NPs. (c) UV–visible absorbance spectra of CTS NPs. (d) Tauc plot of CTS NPs.

Figure 1a shows the XRD pattern of the synthesized CTS NPs. The peaks appearing at 2θ = 28.50, 32.9, 47.3, 56.6, 68.9, and 76.3° correspond to the (111), (200), (220), (311), (400), and (331) crystal planes of CTS, respectively. The diffraction pattern is in good agreement with the standard JCPDS data (No. 89-2877) for the cubic phase of CTS. The narrow and sharp peaks indicate good crystallinity, and the results are well matched with previous literature reports.^46^ The observed highest peak intensity for the (111) plane indicates that CTS prefers growth in this direction. The average crystallite size is estimated using the Scherrer equation, *D* = 51 nm *k*λ/βcosθ,^47^ where *D* is crystalline size, λ is the wavelength of incident X-ray, *k* is the shape factor, θ is the Bragg angle, and β is the full width at half-maximum (FWHM). Due to the polycrystalline nature of CTS, it can stabilize in various crystal phases like tetragonal, cubic, and monoclinic, all of which exhibit a similar XRD pattern.^48^ Hence, in addition to XRD structural analysis, we have used Raman spectroscopy to confirm the formation of the pure cubic CTS phase, as it is sensitive to lattice vibration and can easily be differentiated from other crystal structures. The Raman spectra of the CTS NPs exhibit two major peaks at 292 and 344 cm^–1^ (Figure 1b), confirming the formation of cubic CTS phase, and they match well with previous reports.^42,49^ As the optical absorption plays a crucial role in determining the suitability of a material for photovoltaic and PEC application, we have recorded the optical absorption of CTS in the wavelength range of 300–1200 nm as shown in Figure 1c and it is consistent with a recent report.^50^ The optical band gap of the CTS NPs was calculated using the Tauc relation α*hυ* = *B*(*hυ* – *E*_g_)*^n^*, where *B* is the Tauc constant, *h* is Plank’s constant, υ is the photon frequency, and *E*_g_ is the band gap of the material. In the Tauc plot shown in Figure 1d, the band gap of CTS NPs is estimated at 1.1 eV, indicating that it can be effectively used as an absorber layer in a photoelectrochemical cell.

### 3.2 Oxidation State and Compositional Analyses of CTS NPs

**Figure 2:**
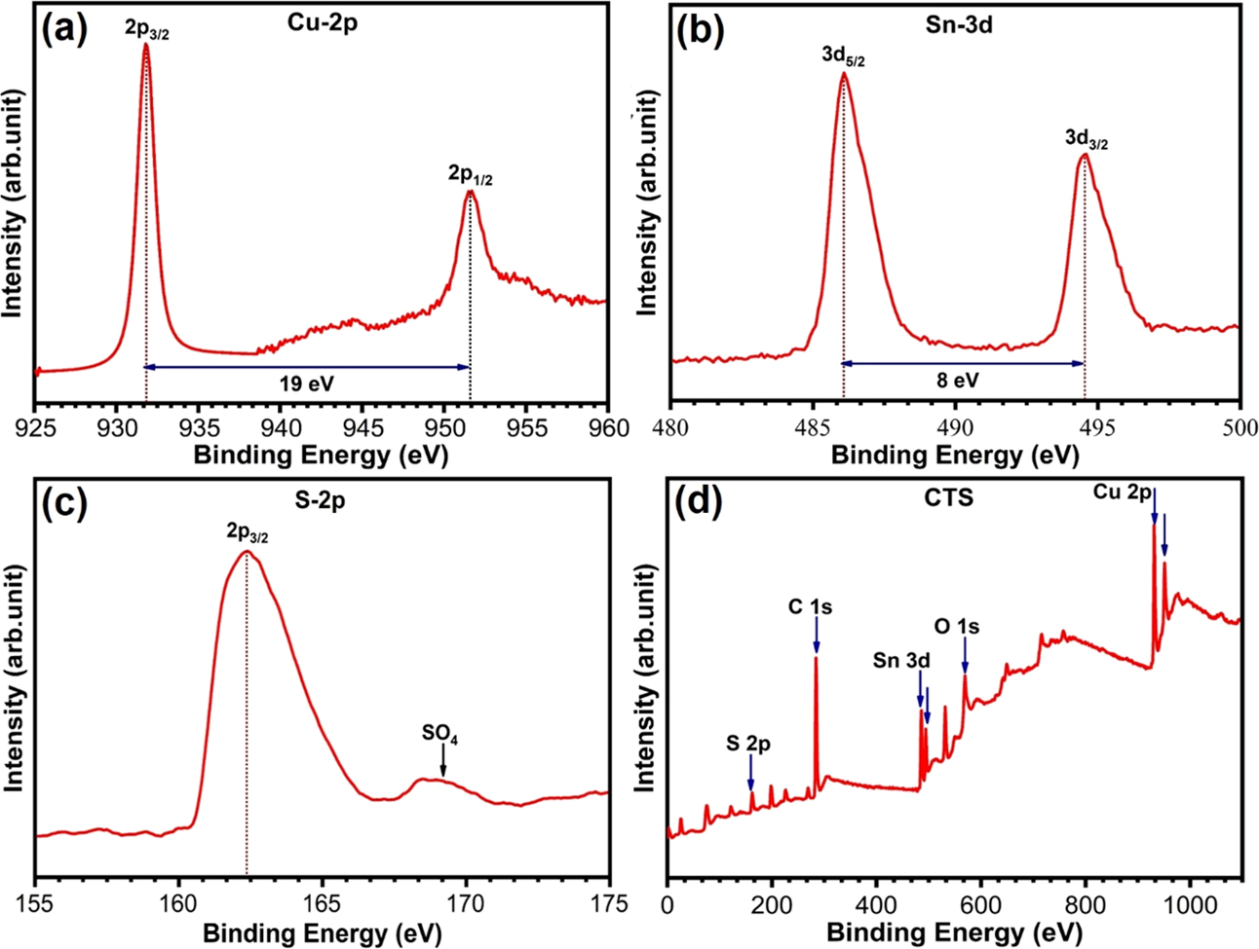
High-resolution XPS spectra of (a) Cu 2p, (b) Sn 3d, (c) S 2p, and (d) survey scan spectrum of CTS NPs.

The oxidation states and composition of the as-prepared CTS NPs were systematically investigated using XPS analysis. Shown in Figure 2a–c are the core-level spectra of Cu 2p, Sn 3d, and S 2p, respectively. The survey spectra in Figure 2d show the presence of Cu, Sn, and S elements with C and O. In Figure 2a, the two major peaks located at binding energies of 931.7 and 951.6 eV correspond to the Cu 2p_3/2_ and Cu 2p_1/2_, which are consistent with the values of Cu^+^ state in CTS.^46^ The energy separation between these two peaks is 19.9 eV. Figure 2b depicts the double peak in the Sn 3d spectrum of CTS, confirming the presence of Sn in CTS. The spin-orbit of Sn 3d_5/2_ and Sn 3d_3/2_ peaks discovered at binding energies of 486.07 and 494.53 eV, respectively, indicates the presence of Sn^4+^ oxidation state species in the CTS crystal structure. The energy separation between these two peaks is 8.46 eV.^51^ The core-level spectrum of S 2p_3/2_ in Figure 2c exhibits a peak located at a lower energy of 162.29 eV, which is attributed to the presence of S^2–^ valence in the CTS composite.^42^ Moreover, the satellite peak located at 169.81 eV suggests the presence of sulfate sulfur formed by the surface oxidation of the CTS NPs.^52^ The peaks of carbon and oxygen present in the survey scan spectrum are due to the ODE solvent and hydrous metal precursor sources used during synthesis. So, it can be concluded that the valence state of Cu_2_SnS_3_ is Cu^+^, Sn^4+^, and S^2–^ , which is also consistent with the XRD results discussed above. EDS composition analysis (Figure S8a) confirmed the existence of Cu, Sn, and S with the chemical composition Cu = 42.97%, Sn = 11.99%, and S = 45.04%, which is near the optimal stoichiometric ratio of Cu_2_SnS_3_. The elemental mapping (Supporting Information, Figure S8b–e) of the cubic CTS NPs shows the homogeneous elemental distribution within the CTS NPs.

**Table 1:** Electrochemical Band Structure Parameters (Valance (CB) and Conduction (CB) Band Edges vs NHE and Vacuum) and Band Gaps (E_g_) Estimated from Cyclic Voltammetry and UV–Vis Spectroscopy in eV Units

sr. no.	sample	VB vs NHE	CB vs NHE	VB vs Vac	CB vs Vac	E_g_ (CV)	E_g_ (UV–vis)
1	CTS	0.51	–0.54	–5.01	–3.96	1.05	1.1
2	CdS	1.86	–0.46	–6.36	–4.04	2.32	2.2
3	ZnS	2.79	–0.83	–7.29	–3.67	3.62	3.6

### 3.3 Surface Morphological Analyses of CTS NPs

**Figure 3:**
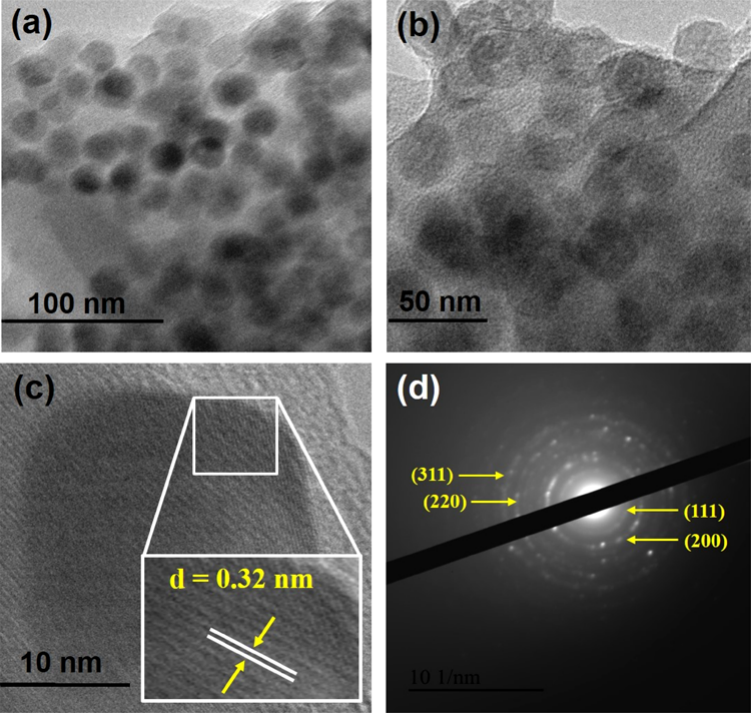
(a & b) Low-resolution TEM images recorded for CTS NPs. (c) HR-TEM images of CTS NPs with clear lattice resolution. (d) Selected area electron diffraction pattern of CTS NPs; corresponding lattice plane is indexed.

Transmission electron microscopy (TEM) images and selected area diffraction pattern (SAED) recorded for the CTS NPs are shown in Figure 3. The TEM image (Figure 3a–b) shows that the CTS nanoparticles possess spherical shapes with diameter ranging from 15 to 25 nm. The HR-TEM images of CTS NPs shown in Figure 3c have lattice fringes with an interplanar distance *d* = 0.32 nm, which can be assigned to the (111) plane, and are in good agreement with the XRD pattern. The concentric ring observed in the SAED pattern **(**Figure 3d) shows the polycrystalline nature of CTS NPs. Figure S9 shows the scanning electron microscopy (SEM) images of the CTS NPs at different magnifications, revealing a non-uniform spherical morphology with an aggregation of particles.

### 3.4 Photoelectrochemical Measurements

The photoelectrochemical (PEC) response of the CTS thin films was studied in a constructed PEC cell by PEIS and M–S analyses in 0.5 M Na_2_SO_4_ at pH 7. For illumination, the solar simulator AM 1.5 G (100 mW cm^–2^) was used and the potentials were converted to NHE and RHE as follows:^53^
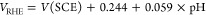
1
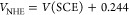
2

**Figure 4:**
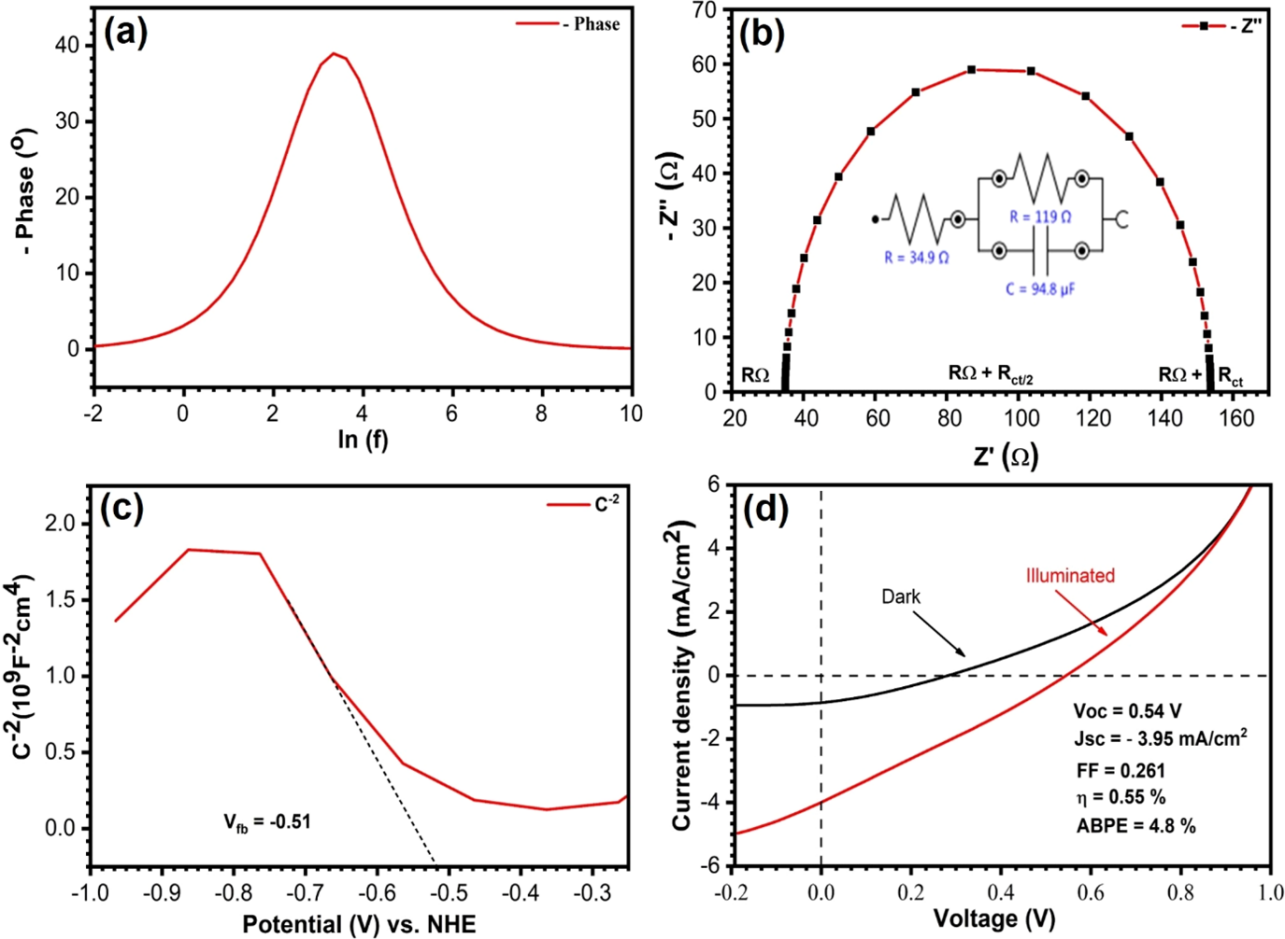
(a) Bode phase plot. (b) Z′ real vs −Z″ imaginary impedance plot (Nyquist plot) of CTS. (c) Mott–Schottky plot. (d) Current density–potential curves of CTS under simulated AM 1.5 G light irradiation in different conditions.

Impedance (Nyquist plot) spectra under illumination were used to elucidate the kinetics of charge transfer process across the CTS–electrolyte interface.^54^ This has been further understood by the change in phase of sinusoidal waves vs frequency, the Bode plot (Figure 4a), which helps to evaluate the lifetime of the charge carriers, an important parameter that influences the PEC activity. The lifetime (τ) of the electrons before recombination assisted by the frequency (*f*) of the maximum phase change was calculated using the equation^61,62^ τ = 1/2π*f* and summarized in Table 2. Figure 4b displays the Nyquist plot obtained by PEIS data of photocathode CTS, which were best fitted to an equivalent circuit model (inset, Figure 4b). It contains capacitance *C* and resistance *R* = *R*_ct_ + *R*_Ω_, where *R*_ct_ is the charge transfer resistance between the photocathode and electrolyte and *R*_Ω_ is the ohmic (series) resistance. The observed *R*_Ω_ of CTS is very small compared to *R*_ct_. The capacitance and resistance can be validated and studied using M–S analysis for the interfacial properties across the junction. M–S plots were processed utilizing the following equations:^25,55^
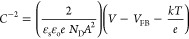
3
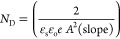
4where ε_o_ is the dielectric constant of the semiconductor, ε_o_is the permittivity of free space, *A* is the area of the thin film, *e* is the charge, and *k* is Boltzmann’s constant. *V*_FB_ indicates the potential required to diffuse the photogenerated charge carriers in the semiconductor. The dielectric constants of CTS (4.8),^56^ CdS (9.35), and ZnS (8.37)^57^ and the slope of the MS plot were used to evaluate the donor concentration (*N*_D_) using eqs 3 and 4, with the results displayed in Table 2. The photostability of the electrode is a very crucial parameter in the long-term use as a device. Figure S10 demonstrates the chronoamperometric photocurrent response of the CTS photoelectrode as a function of time (up to 1000 s). The CTS photocathode is quite stable under dark and light conditions up to 1000 s with an applied bias of −0.5 V. Evaluated parameters of the PEC activities of the photocathode explain the current density obtained by the CTS thin film shown in Figure 4d. The PEC cell energy conversion efficiency (η) for the present CTS film is found to be 0.55% with 0.261 fill factor (FF), −3.95 mA/cm^2^ photocurrent density, and 0.54 V Voc. There is, however, an enormous opportunity to increase the device efficiency through material synthesis and device architecture optimization in the future.

**Table 2:** PEC Data of CTS, CdS, and ZnS, i.e., Resistances (*R*_Ω,_*R*_ct_), Capacitance (*C*), Flat Band Potential (*V*_FB_), Short Circuit Current Density (Jsc), Carrier Lifetime (τ), Donor Concentration (*N*_D_), and ABPE %

sample	*R*_Ω_ (Ω)	*R*_ct_ (Ω)	*C*	*V*_FB_ (V)	ε_s_	*N*_D_ (cm^–3^)	*f*_max_ (kHz)	τ	Jsc	ABPE %
CTS	34.9	119	94.8 μF	–0.51	4.8	4.03 × 10^18^	3.37	5.31 ms	–3.95 mA	4.8
CdS	2.94	2131.69	8.01 μF	–0.46	9.35	8.03 × 10^16^	59.87	2.65 μs	0.32 mA	0.39
ZnS	0.23	380.34	0.12 nF	–0.39	8.37	6.54 × 10^16^	35.95	4.42 μs	0.29 μA	3.5 × 10^–4^

**Scheme 2:**
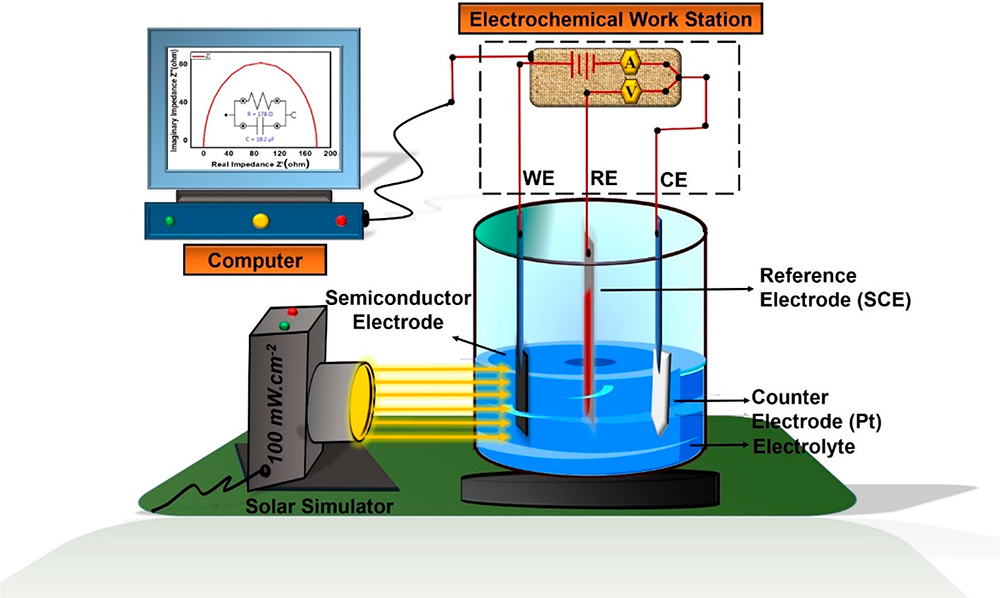
Photoelectrochemical (PEC) Measurement Setup for CTS, CdS, and ZnS Thin Films

Considering that semiconductor heterojunction design strategies are effective to promote the efficient separation charge carriers and minimize their recombination,^58^ we expect that further performance enhancement can be achieved through coupling of CTS with a suitable n-type semiconductor to the formed heterojunction. The beneficial role of n-type material heterointerface (p–n junction) with p-type CTS for low-cost photovoltaics, gas sensing, and photoelectrochemical sensor applications has been reported.^59−62^ Here, we propose CdS and ZnS as suitable photoanode materials and hence quantified their structural, optical, and PEC activities. The XRD pattern and Raman spectrum of the CdS (Figure S11a,b) and ZnS (Figure S12a,b) NPs confirm their crystallinity and phase purity. The Raman spectrum of the CdS NPs (Figure S11b) shows two sharp peaks at 300 and 600 cm^–1^, which correspond to the 1LO and 2LO phonon modes of phase-pure CdS.^63^ FTIR results (Figure S12b) show that the ZnS NPs were successfully capped with the oleylamine ligand. The peak at 617 cm^–1^ corresponds to the metal–sulfur vibrations present in the ZnS NPs.^64^ The band gap of CdS and ZnS NPs is estimated at 2.3 and 3.6 eV from the Tauc plots shown in Figures S11d and S12d, respectively. In the Supporting Information, Figure S13d for CdS and Figure S14d for ZnS confirm their characteristic n-type semiconducting behavior, *i.e.*, photoanodic behavior, with CdS exhibiting a higher current density response than ZnS. At 0 *V*_RHE_, ZnS gives a negative Jsc, and after 0.28 V_RHE_, it has a photoanodic behavior. In addition, the applied bias photon-to-current efficiency (ABPE)^53,55^ evaluated for CTS, CdS, and ZnS is displayed in Table 2 and supports CdS to be a better photoanode to create a heterojunction with CTS. Scheme 2 represents the photoelectrochemical measurement setup for CTS, CdS, and ZnS thin films.

### 3.5 Cyclic Voltammetric Measurements

**Figure 5:**
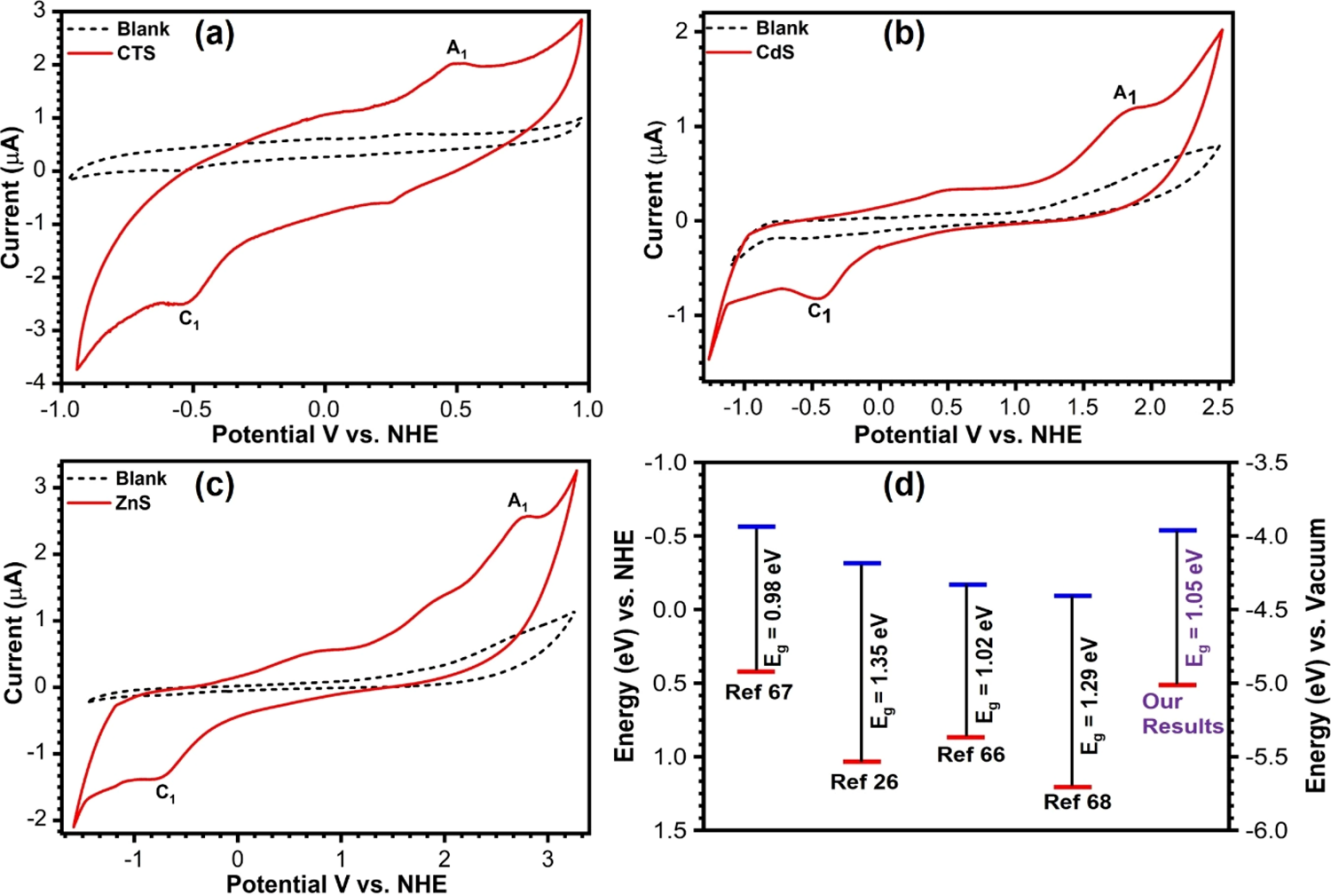
Cyclic voltammogram for CTS (a), CdS (b), and ZnS (c) recorded on drop casted GC at a scan rate 100 mv/s. The black dotted line is for the blank electrolyte, and the solid red line is for samples. (d) Band edge positions of CTS for the present investigation and from the previous literature are compared and demonstrated vs NHE and vacuum.

As information regarding the band offsets and alignment at the buffer–absorber interface is necessary for the optimization of heterojunctions to achieve enhanced PEC activities, we have employed a combination of state-of-the-art material simulation techniques and cyclic voltammetric (CV) experiments to determine the band alignment of CTS/CdS and CTS/ZnS heterojunctions. All the CV measurements were performed in an argon atmosphere in DCM solvent with TBAP as the supporting electrolyte. The cyclic voltammograms for CTS, CdS, and ZnS are shown in Figure 5a–c. Controlled CVs were recorded on the bare GC electrode without any sample loading and shown as dotted black lines, while a solid red line represents the GC electrode with 100 μL of sample loaded. The prominent anodic (A_1_) and cathodic (C_1_) peaks are obtained at 0.51 and −0.54 V, respectively, for CTS, 1.88 and −0.46 V for CdS, and 2.79 and 0.83 V for ZnS. Specifically, in the electrochemical processes taking place at the semiconductor electrode/electrolyte interface, the A_1_ peak potential (*i.e.*, oxidation = loss of electron) corresponds to the removal of electron from the VB edge, and the C_1_ peak potential (*i.e.*, reduction = gain of electron) represents the addition of electron to the CB edge of semiconducting nanocrystals. Therefore, the values of electron affinities and ionization potential can be directly deduced from A_1_ and C_1_, respectively. Thus, the observed A_1_ and C_1_ peaks in the CVs are attributed to the electron transfer via the valence and conduction band edges, respectively. The potential differences between peaks A_1_ and C_1_ (*i.e.*, electrochemical band gap) for CTS, CdS, and ZnS are 1.05, 2.32, and 3.62 V, which coincide with the optical band gaps of 1.1, 2.2, and 3.6 eV, respectively, calculated from the UV–vis spectroscopy (see Table 1). The valence band edge and conduction band edge positions estimated with respect to NHE and local vacuum for CTS, CdS, and ZnS NPs are given in Table 1. Figure 5d shows the electronic band edge parameters for CTS samples available in the literature^26,65−67^ compared with our results. Jia *et al*. used the XPS-based valence band spectra analysis to estimate the valence band edge and took the difference between the optical band gap and valence band to assume the conduction band edge for the CTS/In_2_S_3_ interface.^66^ Dias *et al*. synthesized CTS quantum dots of varied sizes (2.7–3.6 nm) as a function of reaction time and observed a variation in the band gap (0.69–1.26 eV). The 3.3 nm quantum dot showed an optical band gap of 1.15 eV, while from CV measurements, the band gap was estimated at 1.02 eV, and these quantum dots were studied for IR photodetector applications.^65^ Shelke *et al.* studied the effect of annealing temperature on the optoelectronic properties of CTS films using chemical bath deposition and obtained a band gap within 1.31–1.35 eV. The conduction band edge positions of the CTS films were estimated from their Mott–Schottky plot.^26^ Similarly, Patel *et al.* studied the electrical properties of CTS thin film prepared by spray pyrolysis with varied Cu concentration and estimated the band gaps (1.29–1.73 eV).^67^ The observed deviations in the band gap and band edge positions of CTS in Figure 5d could be attributed to differences in synthesis method, size, shapes, and more importantly techniques used for the estimation of the band edges.

### 3.6 Band Alignment of CTS/CdS and CTS/ZnS Heterojunctions

**Figure 6:**
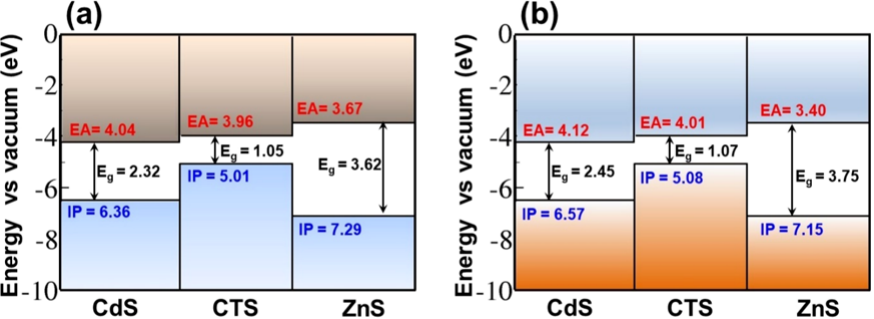
Schematic energy band diagram of p-Cu_2_SnS_3_/n-CdS and p-Cu_2_SnS_3_/n-ZnS heterojunctions based on (a) measured and (b) DFT calculated electron affinity (CBM) and ionization potential (VBM) of Cu_2_SnS_3_, CdS, and ZnS with respect to the vacuum level.

The valence and conduction band edges of the CTS, CdS, and ZnS were determined by CV measurements. These measurements permit the estimation of the electrochemical ionization potential (IP) and the electron affinity (EA). On the basis of the estimated IP and EA values, the band alignment of the CTS/CdS and CTS/ZnS heterojunctions was constructed and is shown in Figure 6a. A staggered gap (type II) band alignment is found at the CTS/CdS interface, whereas a straddling gap (type I) band alignment is observed at the CTS/ZnS interface. The conduction band offset (CBO) at the CTS/CdS and CTS/ZnS interface is estimated at 0.08 and 0.29 eV, respectively. The conduction band minimum (CBM) of CTS was found to be higher than that of CdS and lower than that of ZnS. The very small conduction band offset (CBO) of 0.08 eV measured at the CTS/CdS heterojunction is an encouraging factor for PEC cell energy conversion efficiency.

**Figure 7:**
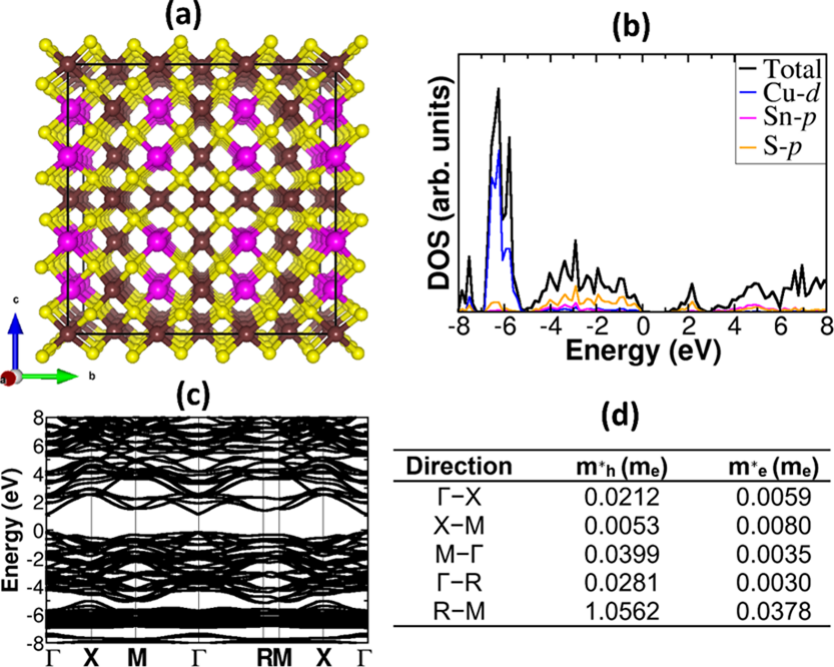
(a) Crystal structure, (b) partial density of states (PDOS), and (c) band structure of CTS Model 1. The calculated corresponding effective masses of holes and electrons along the high-symmetry directions of the Brillouin zone are shown in (d). Atomic color: Cu = brown, Sn = pink, and S = yellow.

Further insights into the electronic structure of CTS, CdS, and ZnS materials and the energy band alignment at the CTS/CdS and CTS/ZnS heterojunctions were gained from first-principles DFT calculations. First, the electronic structures (partial density of states (PDOS) and band structure) of the CTS Model 1 (Figure 7b,c), ZnS (Figure S3b,c), and CdS (Figure S4b,c) materials were determined using the screened hybrid HSE06 functional,^37^ predicting direct band gaps of 1.07, 2.45, and 3.75 eV, respectively. The predicted band gap for CTS in Model 1 is in good agreement with our UV–vis spectroscopy value of 1.1 eV and the electrochemical value of 1.05 eV. These are also consistent with the band gap reported for bulk CTS crystals (0.94 eV)^68^ and CTS nanoparticles as well as CTS thin films (0.92–1.02 eV).^18,21,69−72^ In contrast, Dias *et al*.^51^ reported a larger band gap of 1.66 eV for CTS quantum dots with a very small size (∼3 nm), which is less than the exciton Bohr radius for CTS.^73^ From the predicted electronic band structures, the effective masses of electrons (*m*_e_^*^) and holes (*m*_h_^*^) for CTS, CdS, and ZnS were calculated by fitting the energy of the conduction band minimum and valence band maximum, respectively, to a quadratic polynomial in the reciprocal lattice vector *k* according to the relations: . The calculated *m*_e_^*^ and *m*_h_^*^ for CTS are shown in Figure 7d, whereas those for ZnS and CdS are provided in the Supporting Information (Figures S3d and S4d). In general, we found that the electrons have lighter effective masses than the holes in CTS, ZnS, and CdS materials, indicating that the electrons are more mobile than the holes. The predicted small effective masses are highly desirable for promoting efficient separation of photogenerated charge carriers in CTS, which is essential for achieving improved photoelectrochemical performance.

The IP and EA of the CTS(111), CdS(100), and ZnS(110) surfaces (Figures S5–S7) were calculated to construct their band energy levels relative to the vacuum level as displayed in Figure 6b. The IP and EA values are predicted at 5.08 and 4.01 eV for CTS(111), 6.57 and 4.12 eV for CdS (100), and 7.15 and 3.40 eV for ZnS(110). Based on the calculated IP and EA values, a staggered type-II and straddling type-I band alignment is predicted to exist at the CTS/CdS and CTS/ZnS heterojunctions, respectively (Figure 6b). There is a good agreement between measured and simulated values of band edge energetics for each material. Most especially, the simulated values reproduce very well the relative energy levels of the VBM and CBM of CTS, CdS, and ZnS materials, which are important for determining the electron/hole transfer directions in the CTS/CdS and CTS/ZnS heterojunctions. Photogenerated conduction electrons are expected to move from the CTS to the CdS and ZnS layers in the CTS/CdS and CTS/ZnS heterostructure, respectively. On the other hand, photogenerated valence band holes will flow to the CTS layer in both CTS/CdS and CTS/ZnS heterojunctions.

## 4 Conclusions

In summary, we have demonstrated a facile and low-cost hot injection approach for the synthesis of phase-pure Cu_2_SnS_3_, CdS, and ZnS NPs as confirmed by XRD and Raman spectroscopy analyses. The structural, morphological, optoelectronic, band edge, and interface properties of the as-prepared materials were comprehensively characterized using state-of-the-art experimental techniques and corroborated by first-principles DFT calculations. The fabricated stable CTS photocathode exhibits PEC cell energy conversion efficiency (η) of 0.55% with 0.26 fill factor (FF), photocurrent density of (Jsc) −3.95 mA/cm^2^, and 0.54 V Voc. CdS, which exhibits a higher current density response than ZnS, is demonstrated to be a better photoanode to create a heterojunction with CTS. Consistently, a staggered type-II band alignment with a small CBO of 0.08 eV is predicted to exist at the CTS/CdS heterojunction, which is promising for efficient charge carrier separation and transport across the interface. The successful preparation of phase-pure CTS NPs and thin films using facile and economical hot injection and spin coating techniques could have immense potential for low-cost and large-area deposition of CTS thin films for next-generation Cu-based solar cells, photoelectrochemical cells, and flexible display devices.

## Supporting Information Available

Three possible model arrangements of Cu and Sn ions at the 4a sites in CTS (Figure S1); partial density of states (PDOS) of CTS models (Figure S2); crystal structure, PDOS, band structure, and effective masses of ZnS and CdS (Figures S3 and S4); geometry optimized surface model of CTS(111), CdS(100), and ZnS(110) surfaces used to align the electronic eigenvalues to the vacuum level (Figures S5–S7); EDS spectra and elemental mapping of CTS NPs (Figure S8); SEM images of CTS NPs (Figure S9); chronoampereometry photocurrent stability test for the CTS thin film under illumination of the AM 1.5 G solar light (Figure S10); X-ray diffraction pattern, Raman spectrum, and UV–vis absorbance spectra of CdS and ZnS NPs (Figures S11 and S12); photoanodic behavior of CdS and ZnS (Figures S13 and S14), and the relaxed structures with the lattice parameters and atomic positions for the bulk structures and for the surface slabs generated (PDF)

## References

[ref1] Dinçer F. The Analysis on Photovoltaic Electricity Generation Status, Potential and Policies of the Leading Countries in Solar Energy. Renewable Sustainable Energy Rev. 2011, 15, 713–720. 10.1016/j.rser.2010.09.026.

[ref2] IEA. Trends 2015 in Photovoltaic Applications - Executive Summary. Rep. IEA-PVPS 2015, 9.

[ref3] Jackson P.; Wuerz R.; Hariskos D.; Lotter E.; Witte W.; Powalla M. Effects of Heavy Alkali Elements in Cu(In,Ga)Se_2_ Solar Cells with Efficiencies up to 22.6%. Phys. Status Solidi RRL 2016, 10, 583–586. 10.1002/pssr.201600199.

[ref4] Green M.; Emery K.; Hishikawa Y.; Warta W.; Dunlop E.; Barkhouse D.; Gunawan O.; Gokmen T.; Todorov T.; Mitzi D. Solar Cell Efficiency Tables (Version 40). Ieee Trans. Fuzzy Syst. 2012, 20, 1114–1129.

[ref5] Tanaka A.; Hirata M. Health Effects of Solar Cell Component Material. Toxicity of Indium Compounds to Laboratory Animals Determined by Intratracheal Instillations. Nihon Eiseigaku Zasshi. 2013, 68, 83–87. 10.1265/jjh.68.83. 23718969

[ref6] Wadia C.; Alivisatos A. P.; Kammen D. M. Materials Availability Expands the Opportunity for Large-Scale Photovoltaics Deployment. Environ. Sci. Technol. 2009, 43, 2072–2077. 10.1021/es8019534. 19368216

[ref7] Vasekar P.; Dhakal T. Thin Film Solar Cells Using Earth-Abundant Materials. In Solar Cells - Research and Application Perspectives; IntechOpen Publishing: 2013; 145–168.

[ref8] Wang W.; Winkler M. T.; Gunawan O.; Gokmen T.; Todorov T. K.; Zhu Y.; Mitzi D. B. Device Characteristics of CZTSSe Thin-Film Solar Cells with 12.6% Efficiency. Adv. Energy Mater. 2014, 4, 1301465–1301469. 10.1002/aenm.201301465.

[ref9] Sun K.; Yan C.; Liu F.; Huang J.; Zhou F.; Stride J. A.; Green M.; Hao X. Over 9% Efficient Kesterite Cu_2_ZnSnS_4_ Solar Cell Fabricated by Using Zn_1–X_Cd_x_S Buffer Layer. Adv. Energy Mater. 2016, 6, 1600046–160051. 10.1002/aenm.201600046.

[ref10] Siebentritt S.; Schorr S. Kesterites- a Challenging Materials for Solar Cell. In Progress In Photovoltaics: Research And Applications; John Wiley & Sons Ltd Publishing: 2012, 20, 512–519.

[ref11] Riha S. C.; Parkinson B. A.; Prieto A. L. Compositionally Tunable Cu_2_ZnSn(S_1–x_Se_x_)_4_ Nanocrystals: Probing the Effect of Se-Inclusion in Mixed Chalcogenide Thin Films. J. Am. Chem. Soc. 2011, 133, 15272–15275. 10.1021/ja2058692. 21882872

[ref12] Katagiri H.; Jimbo K.; Maw W. S.; Oishi K.; Yamazaki M.; Araki H.; Takeuchi A. Development of CZTS-Based Thin Film Solar Cells. Thin Solid Films 2009, 517, 2455–2460. 10.1016/j.tsf.2008.11.002.

[ref13] Grenet L.; Suzon M. A. A.; Emieux F.; Roux F. Analysis of Failure Modes in Kesterite Solar Cells. ACS Appl. Energy Mater. 2018, 1, 2103–2113. 10.1021/acsaem.8b00194.

[ref14] Fontané X.; Calvo-Barrio L.; Izquierdo-Roca V.; Saucedo E.; Pérez-Rodriguez A.; Morante J. R.; Berg D. M.; Dale P. J.; Siebentritt S. In-Depth Resolved Raman Scattering Analysis for the Identification of Secondary Phases: Characterization of Cu_2_ZnSnS_4_ Layers for Solar Cell Applications. Appl. Phys. Lett. 2011, 98, 181905–181907. 10.1063/1.3587614.

[ref15] Kumar M.; Dubey A.; Adhikari N.; Venkatesan S.; Qiao Q. Strategic Review of Secondary Phases, Defects and Defect-Complexes in Kesterite CZTS-Se Solar Cells. Energy Environ. Sci. 2015, 8, 3134–3159. 10.1039/C5EE02153G.

[ref16] Chen F.; Zai J.; Xu M.; Qian X. 3D-Hierarchical Cu_3_SnS_4_ Flowerlike Microspheres: Controlled Synthesis, Formation Mechanism and Photocatalytic Activity for H_2_ Evolution from Water. J. Mater. Chem. A 2013, 1, 4316–4323. 10.1039/c3ta01491f.

[ref17] Xu J.; Yang X.; Wong T. L.; Lee C. S. Large-Scale Synthesis of Cu_2_SnS_3_ and Cu_1.8_S Hierarchical Microspheres as Efficient Counter Electrode Materials for Quantum Dot Sensitized Solar Cells. Nanoscale 2012, 4, 6537–6542. 10.1039/c2nr31724a. 22968176

[ref18] Berg D. M.; Djemour R.; Gütay L.; Zoppi G.; Siebentritt S.; Dale P. J. Thin Film Solar Cells Based on the Ternary Compound Cu_2_SnS_3_. Thin Solid Films 2012, 520, 6291–6294. 10.1016/j.tsf.2012.05.085.

[ref19] Zawadzki P.; Baranowski L. L.; Peng H.; Toberer E. S.; Ginley D. S.; Tumas W.; Zakutayev A.; Lany S. Evaluation of Photovoltaic Materials within the Cu-Sn-S Family. Appl. Phys. Lett. 2013, 103, 253902–253907. 10.1063/1.4851896.

[ref20] Kuku T. A.; Fakolujo O. A. Photovoltaic Characteristics of Thin Films of Cu_2_SnS_3_. Sol. Energy Mater. 1987, 16, 199–204. 10.1016/0165-1633(87)90019-0.

[ref21] Nakashima M.; Fujimoto J.; Yamaguchi T.; Izaki M. Cu_2_SnS_3_ Thin-Film Solar Cells Fabricated by Sulfurization from NaF/Cu/Sn Stacked Precursor. Appl. Phys. Express 2015, 8, 042303–042307. 10.7567/APEX.8.042303.

[ref22] Umehara M.; Takeda Y.; Motohiro T.; Sakai T.; Awano H.; Maekawa R. Cu_2_Sn_1-X_Ge_x_S_3_ (x = 0:17) Thin-Film Solar Cells with High Conversion Efficiency of 6.0%. Appl. Phys. Express 2013, 6, 045501–045504. 10.7567/APEX.6.045501.

[ref23] Avellaneda D.; Nair M. T. S.; Nair P. K. CuZ_2_SnS_3_ and Cu_4_SnS_4_ Thin Films via Chemical Deposition for Photovoltaic Application. J. Electrochem. Soc. 2010, 157, D346–D352. 10.1149/1.3384660.

[ref24] Shelke H. D.; Patil A. M.; Lokhande A. C.; Kim J. H.; Lokhande C. D. Electrochemical Impedance Analysis of SILAR Deposited Cu_2_SnS_3_ ( CTS ) Thin Film. Int. J. Eng. Res. Technol. 2017, 10, 578–586.

[ref25] Shelke H. D.; Lokhande A. C.; Raut V. S.; Patil A. M.; Kim J. H.; Lokhande C. D. Facile Synthesis of Cu_2_SnS_3_ Thin Films Grown by SILAR Method: Effect of Film Thickness. J. Mater. Sci.: Mater. Electron. 2017, 28, 7912–7921. 10.1007/s10854-017-6492-7.

[ref26] Shelke H. D.; Lokhande A. C.; Kim J. H.; Lokhande C. D. Photoelectrochemical (PEC) Studies on Cu_2_SnS_3_ (CTS) Thin Films Deposited by Chemical Bath Deposition Method. J. Colloid Interface Sci. 2017, 506, 144–153. 10.1016/j.jcis.2017.07.032. 28735188

[ref27] Rondiya S.; Wadnerkar N.; Jadhav Y.; Jadkar S.; Haram S.; Kabir M. Structural, Electronic, and Optical Properties of Cu_2_NiSnS_4_: A Combined Experimental and Theoretical Study toward Photovoltaic Applications. Chem. Mater. 2017, 29, 3133–3142. 10.1021/acs.chemmater.7b00149.

[ref28] Rondiya S.; Rokade A.; Sharma P.; Chaudhary M.; Funde A.; Jadhav Y.; Haram S.; Pathan H.; Jadkar S. CZTS/CdS: Interface Properties and Band Alignment Study towards Photovoltaic Applications. J. Mater. Sci.: Mater. Electron. 2018, 29, 4201–4210. 10.1007/s10854-017-8365-5.

[ref29] Jadhav Y. A.; Thakur P. R.; Haram S. K. Data in Brief CZTS_x_Se_1-x_ Nanocrystals : Composition Dependent Method of Preparation , Morphological Characterization and Cyclic Voltammetry Data Analysis. Data Br. 2016, 8, 1072–1079. 10.1016/j.dib.2016.07.026. PMC4970545 27508267

[ref30] Rondiya S. R.; Jadhav Y.; Dzade N. Y.; Ahammed R.; Goswami T.; De Sarkar A.; Jadkar S.; Haram S.; Ghosh H. N. Experimental and Theoretical Study into Interface Structure and Band Alignment of the Cu_2_Zn_1-x_Cd_x_SnS_4_ Heterointerface for Photovoltaic Applications. ACS Appl. Energy Mater. 2020, 3, 5153–5162. 10.1021/acsaem.9b02314. 32905359 PMC7469238

[ref31] Ding Z.; Quinn B. M.; Haram S. K.; Pell L. E.; Korgel B. A.; Bard A. J. Electrochemistry and Electrogenerated Chemiluminescence from Silicon Nanocrystal Quantum Dots. Science 2002, 296, 1293–1297. 10.1126/science.1069336. 12016309

[ref32] Kresse G.; Furthmüller J.; Hafner J. Theory of the Crystal Structures of Selenium and Tellurium: The Effect of Generalized-Gradient Corrections to the Local-Density Approximation. Phys. Rev. B 1994, 50, 13181–13185. 10.1103/PhysRevB.50.13181. 9975508

[ref33] Kresse G.; Joubert D. From Ultrasoft Pseudopotentials to the Projector Augmented-Wave Method. Phys. Rev. B 1999, 59, 1758–1775. 10.1103/PhysRevB.59.1758.

[ref34] Kresse G.; Furthmüller J. Efficient Iterative Schemes for Ab Initio Total-Energy Calculations Using a Plane-Wave Basis Set. Phys. Rev. B 1996, 54, 11169–11186. 10.1103/PhysRevB.54.11169. 9984901

[ref35] Blöchl P. E. Projector Augmented-Wave Method. Phys. Rev. B 1994, 50, 17953–17979. 10.1103/PhysRevB.50.17953. 9976227

[ref36] Perdew J. P.; Burke K.; Ernzerhof M. Generalized Gradient Approximation Made Simple. Phys. Rev. Lett. 1996, 77, 3865 10.1103/PhysRevLett.77.3865. 10062328

[ref37] Krukau A. V.; Vydrov O. A.; Izmaylov A. F.; Scuseria G. E. Influence of the Exchange Screening Parameter on the Performance of Screened Hybrid Functionals. J. Chem. Phys. 2006, 125, 224106–224111. 10.1063/1.2404663. 17176133

[ref38] Blöchl P. E.; Jepsen O.; Andersen O. K. Improved Tetrahedron method for Brillouin-zone integrations. Phys. Rev. B 1994, 49, 16223–16233. 10.1103/PhysRevB.49.16223. 10010769

[ref39] Pack J. D.; Monkhorst H. J. “special Points for Brillouin-Zone Integrations”-a Reply. Phys. Rev. B 1977, 16, 1748–1749. 10.1103/PhysRevB.16.1748.

[ref40] Zhao H.; Xu X.; Li C.; Tian R.; Zhang R.; Huang R.; Lyu Y.; Li D.; Hu X.; Pan L.; Wang Y. Cobalt-Doping in Cu_2_SnS_3_: Enhanced Thermoelectric Performance by Synergy of Phase Transition and Band Structure Modification. J. Mater. Chem. A 2017, 5, 23267–23275. 10.1039/C7TA07140J.

[ref41] Minnam Reddy V. R.; Pallavolu M. R.; Guddeti P. R.; Gedi S.; Yarragudi Bathal Reddy K. K.; Pejjai B.; Kim W. K.; Kotte T. R. R.; Park C. Review on Cu_2_SnS_3_, Cu_3_SnS_4_, and Cu_4_SnS_4_ Thin Films and Their Photovoltaic Performance. J. Ind. Eng. Chem. 2019, 76, 39–74. 10.1016/j.jiec.2019.03.035.

[ref42] Lokhande A. C.; Pawar S. A.; Jo E.; He M.; Shelke A.; Lokhande C. D.; Kim J. H. Amines Free Environmentally Friendly Rapid Synthesis of Cu_2_SnS_3_ Nanoparticles. Opt. Mater. 2016, 58, 268–278. 10.1016/j.optmat.2016.03.032.

[ref43] Yang R. X.; Butler K. T.; Walsh A. Assessment of Hybrid Organic-Inorganic Antimony Sulfides for Earth-Abundant Photovoltaic Applications. J. Phys. Chem. Lett. 2015, 6, 5009–5014. 10.1021/acs.jpclett.5b02555. 26624204

[ref44] Burton L. A.; Walsh A. Band Alignment in SnS Thin-Film Solar Cells: Possible Origin of the Low Conversion Efficiency. Appl. Phys. Lett. 2013, 102, 132111–132114. 10.1063/1.4801313.

[ref45] Walsh A.; Butler K. T. Prediction of Electron Energies in Metal Oxides. Acc. Chem. Res. 2013, 47, 364–372. 10.1021/ar400115x. 24066925

[ref46] Wang C.; Tian H.; Jiang J.; Zhou T.; Zeng Q.; He X.; Huang P.; Yao Y. Facile Synthesis of Different Morphologies of Cu_2_SnS_3_ for High-Performance Supercapacitors. ACS Appl. Mater. Interfaces 2017, 9, 26038–26044. 10.1021/acsami.7b07190. 28737372

[ref47] William D.; Callister J.; Rethwisch D. G. Applications and Processing of Ceramics. Mater. Sci. Eng. 2013, 536.

[ref48] Nomura T.; Maeda T.; Takei K.; Morihama M.; Wada T. Crystal Structures and Band-Gap Energies of Cu_2_Sn(S,Se)_3_ (0≤ x ≤1.0) Solid Solution. Phys. Status Solidi C 2013, 10, 1093–1097. 10.1002/pssc.201200867.

[ref49] Wang W.; Shen H.; Li J. Rapid Synthesis of Hollow CTS Nanoparticles Using Microwave Irradiation. Mater. Lett. 2013, 111, 5–8. 10.1016/j.matlet.2013.08.038.

[ref50] Ghorpade U. V.; Suryawanshi M. P.; Shin S. W.; Kim I.; Ahn S. K.; Yun J. H.; Jeong C.; Kolekar S. S.; Kim J. H. Colloidal Wurtzite Cu_2_SnS_3_ (CTS) Nanocrystals and Their Applications in Solar Cells. Chem. Mater. 2016, 28, 3308–3317. 10.1021/acs.chemmater.6b00176.

[ref51] Dias S.; Kumawat K.; Biswas S.; Krupanidhi S. B. Solvothermal Synthesis of Cu_2_SnS_3_ Quantum Dots and Their Application in Near-Infrared Photodetectors. Inorg. Chem. 2017, 56, 2198–2203. 10.1021/acs.inorgchem.6b02832. 28182411

[ref52] Nakai I.; Sugitani Y.; Nagashima K.; Niwa Y. X-Ray Photoelectron Spectroscopic Study of Copper Minerals. J. Inorg. Nucl. Chem. 1978, 40, 789–791. 10.1016/0022-1902(78)80152-3.

[ref53] Liu Z.; Li C.; Xiao Y.; Wang F.; Yu Q.; Faheem M. B.; Zhou T.; Li Y. Tailored NiFe Catalyst on Silicon Photoanode for Efficient Photoelectrochemical Water Oxidation. J. Phys. Chem. C 2020, 124, 2844–2850. 10.1021/acs.jpcc.9b10967.

[ref54] Yun T. K.; Cheon J. H.; Bae J. Y.; Ahn K. S.; Kim J. H. Enhanced Electron Lifetime on Nitrogen-Doped TiO_2_ Films for Dye-Sensitized Solar Cells. J. Nanosci. Nanotechnol. 2012, 12, 3305–3308. 10.1166/jnn.2012.5567. 22849112

[ref55] Dey K. K.; Gahlawat S.; Ingole P. P. BiVO_4_ Optimized to Nano-Worm Morphology for Enhanced Activity towards Photoelectrochemical Water Splitting. J. Mater. Chem. A 2019, 7, 21207–21221. 10.1039/C9TA07353A.

[ref56] Bouaziz M.; Amlouk M.; Belgacem S. Structural and Optical Properties of Cu_2_SnS_3_ Sprayed Thin Films. Thin Solid Films 2009, 517, 2527–2530. 10.1016/j.tsf.2008.11.039.

[ref57] Young K. F.; Frederikse H. P. R. Compilation of the Static Dielectric Constant of Inorganic Solids. J. Phys. Chem. Ref. Data 1973, 2, 313–410. 10.1063/1.3253121.

[ref58] Wang H.; Zhang L.; Chen Z.; Hu J.; Li S.; Wang Z.; Liu J.; Wang X. Semiconductor Heterojunction Photocatalysts: Design, Construction, and Photocatalytic Performances. Chem. Soc. Rev. 2014, 43, 5234–5244. 10.1039/C4CS00126E. 24841176

[ref59] Dias S.; Murali B.; Krupanidhi S. B. Transport Properties of Solution Processed Cu_2_SnS_3_/AZnO Heterostructure for Low Cost Photovoltaics. Sol. Energy Mater. Sol. Cells 2015, 143, 152–158. 10.1016/j.solmat.2015.06.046.

[ref60] Lokhande A. C.; Yadav A. A.; Lee J. Y.; He M.; Patil S. J.; Lokhande V. C.; Lokhande C. D.; Kim J. H. Room Temperature Liquefied Petroleum Gas Sensing Using Cu_2_SnS_3_/CdS Heterojunction. J. Alloys Compd. 2017, 709, 92–103. 10.1016/j.jallcom.2017.03.135.

[ref61] Wang Q.; Zhou M.; Zhang L. A Dual Mode Photoelectrochemical Sensor for Nitrobenzene and L-Cysteine Based on 3D Flower-like Cu_2_SnS_3_@SnS_2_ Double Interfacial Heterojunction Photoelectrode. J. Hazard. Mater. 2020, 382, 121026–121035. 10.1016/j.jhazmat.2019.121026. 31446355

[ref62] Tiwari D.; Chaudhuri T. K.; Shripathi T.; Deshpande U.; Rawat R. Non-Toxic, Earth-Abundant 2% Efficient Cu_2_SnS_3_ Solar Cell Based on Tetragonal Films Direct-Coated from Single Metal-Organic Precursor Solution. Sol. Energy Mater. Sol. Cells 2013, 113, 165–170. 10.1016/j.solmat.2013.02.017.

[ref63] Kamble M. M.; Rondiya S. R.; Bade B. R.; Kore K. B.; Nasane M. P.; Dzade N. Y.; Funde A. M.; Jadkar S. R. Optical, Structural and Morphological Study of CdS Nanoparticles: Role of Sulfur Source. Nanomater. Energy 2020, 9, 72–81. 10.1680/jnaen.19.00041.

[ref64] Rao M. D.; Pennathur G. Facile Bio-Inspired Synthesis of Zinc Sulfide Nanoparticles Using Chlamydomonas Reinhardtii Cell Free Extract: Optimization, Characterization and Optical Properties. Green Process. Synth. 2016, 5, 379–388. 10.1515/gps-2016-0008.

[ref65] Dias S.; Kumawat K. L.; Biswas S.; Krupanidhi S. B. Heat-up Synthesis of Cu_2_SnS_3_ Quantum Dots for near Infrared Photodetection. RSC Adv. 2017, 7, 23301–23308. 10.1039/C7RA02485A. 28182411

[ref66] Jia H.; Cheng S.; Zhang H.; Yu J.; Lai Y. Band Alignment at the Cu_2_SnS_3_/In_2_S_3_ Interface Measured by X-Ray Photoemission Spectroscopy. Appl. Surf. Sci. 2015, 353, 414–418. 10.1016/j.apsusc.2015.06.101.

[ref67] Patel B.; Pati R. K.; Mukhopadhyay I.; Ray A. Electrical Properties Modulation in Spray Pyrolysed Cu_2_SnS_3_ Thin Films through Variation of Copper Precursor Concentration for Photovoltaic Application. J. Anal. Appl. Pyrolysis 2018, 136, 35–43. 10.1016/j.jaap.2018.11.003.

[ref68] Aihara N.; Matsumoto Y.; Tanaka K. Exciton Luminescence from Cu_2_SnS_3_ Bulk Crystals. Appl. Phys. Lett. 2016, 108, 092107–092111. 10.1063/1.4943229.

[ref69] Okano S.; Takeshita S.; Isobe T. Formation of Cu_2_SnS_3_ Nanoparticles by Sequential Injection of Tin and Sulfur Oleylamine Solutions into Cu1.8S Nanoparticle Dispersion. Mater. Lett. 2015, 145, 79–82. 10.1016/j.matlet.2015.01.047.

[ref70] Chang J.; Waclawik E. R. Controlled Synthesis of CuInS_2_, Cu_2_SnS_3_ and Cu_2_ZnSnS_4_ Nano-Structures: Insight into the Universal Phase-Selectivity Mechanism. CrystEngComm 2013, 15, 5612–5619. 10.1039/c3ce40284c.

[ref71] Li B.; Xie Y.; Huang J.; Qian Y. Synthesis Characterization, and Properties of Nanocrystalline Cu_2_SnS_3_. J. Solid State Chem. 2000, 153, 170–173. 10.1006/jssc.2000.8772.

[ref72] Aihara N.; Araki H.; Takeuchi A.; Jimbo K.; Katagiri H. Fabrication of Cu_2_SnS_3_ thin films by sulfurization of evaporated Cu-Sn precursors for solar cells. Phys. Status Solidi C 2013, 10, 1086–1092. 10.1002/pssc.201200866.

[ref73] Ahamed M. I.; Kumar K. S. Modelling of Electronic and Optical Properties of Cu_2_SnS_3_ Quantum Dots for Optoelectronics Applications. Mater. Sci. Pol. 2019, 37, 108–115. 10.2478/msp-2018-0103.

