# Supplementary material for: Ternary Cu2SnS3: Synthesis, Structure, Photoelectrochemical Activity, and Heterojunction Band Offset and Alignment
Source: Chem Mater. 2021 Mar 3;33(6):1983–93. doi: 10.1021/acs.chemmater.0c03223 (PMC8026117; doi:10.1021/acs.chemmater.0c03223)
Supplement: Supplementary file 1 — cm0c03223_si_001.pdf [file cm0c03223_si_001.pdf]

Supporting Information

Ternary Cu_2SnS_3: Synthesis, Structure, Photoelectrochemical Activity, and Heterojunction Band Offset and Alignment

Sagar B. Jathar[†], Sachin R. Rondiya[§*], Yogesh A. Jadhav[†], Dhanaraj S. Nilegave[†], Russell W. Cross[§], Sunil V. Barma[†], Mamta P. Nasane[†], Shankar A. Gaware[†], Bharat R. Bade[ǂ], Sandesh R. Jadkar[ǂ], Adinath M. Funde[†*], Nelson Y. Dzade[§*]

[†]School of Energy Studies, Savitribai Phule Pune University, Pune 411007, India
[§]School of Chemistry, Cardiff University, Main Building, Park Place, Cardiff, CF10 3AT, Wales, United Kingdom
[ǂ]Department of Physics, Savitribai Phule Pune University, Pune 411007, India

Corresponding Author: RondiyaS@cardiff.ac.uk (SRR), adinathf@gmail.com (AMF), DzadeNY@cardiff.ac.uk (NYD)

The Supporting Information contains 14 Figures (three possible model arrangement of Cu and Sn ions at the 4a sites in CTS; partial density of states (PDOS) of CTS models; crystal structure, PDOS, band structure and effective masses of ZnS and CdS; geometry optimized surface model of CTS(111), CdS(100), and ZnS(110) surfaces used to align the electronic eigenvalues to the vacuum level; EDX spectra and elemental mapping of CTS NPs; SEM images of CTS NPs; chronoampereometry photocurrent stability test for the CTS thin film under illumination of the AM1.5G solar light; X-ray diffraction pattern, Raman spectrum and UV-vis absorbance spectra of CdS and ZnS NPs; and photoanodic behaviour of CdS and ZnS NPs). The relaxed structures with the lattice parameters and atomic positions for the bulk structures, and for the surface slabs generated are also given.

Figure S1: Three possible arrangement of Cu and Sn ions at the 4a sites in CTS: (a) Model-1, (b) Model-2, and (c) Model-3. Model-1 is energetically more favourable than Model-2 by 4.54 eV and Model-3 by 11.98 eV. Atomic color: Cu =brown, Sn =pink and S =yellow.

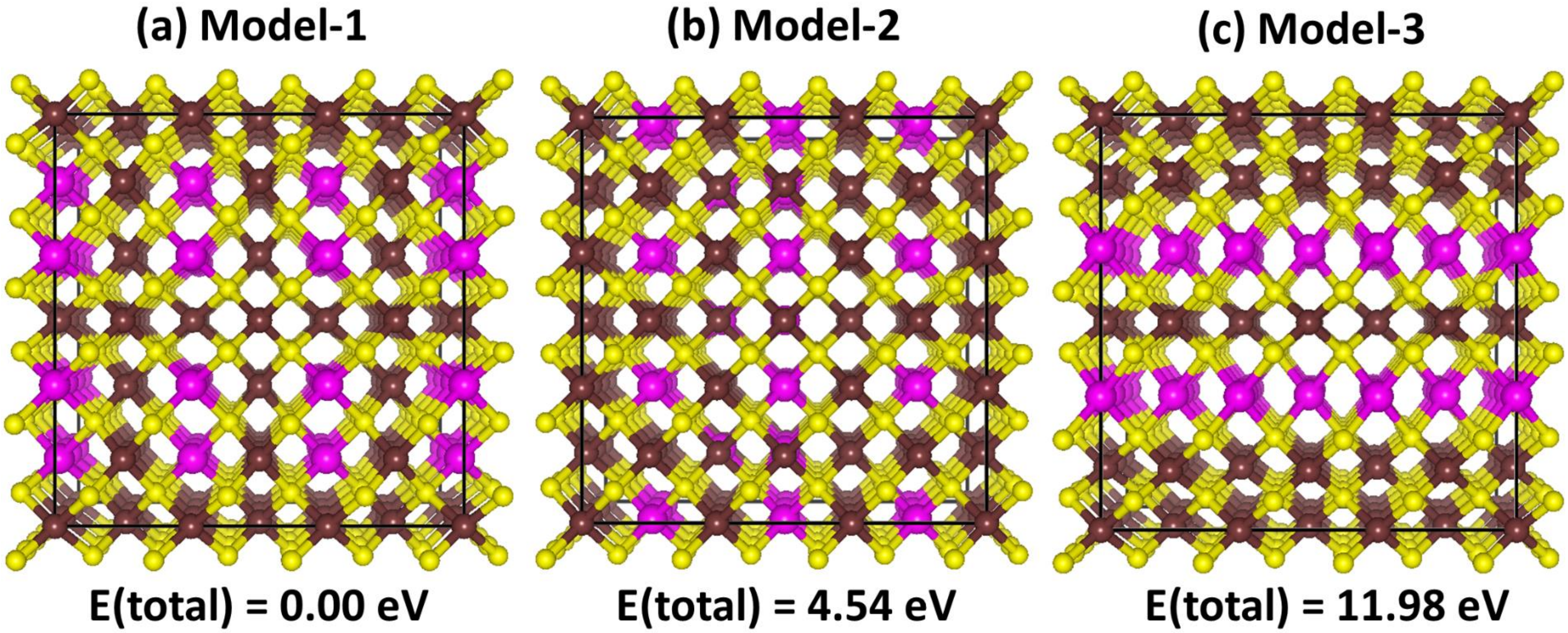

Figure S2: The partial density of states of CTS in (a) Model-1, (b) Model-2, and (c) Model-3.

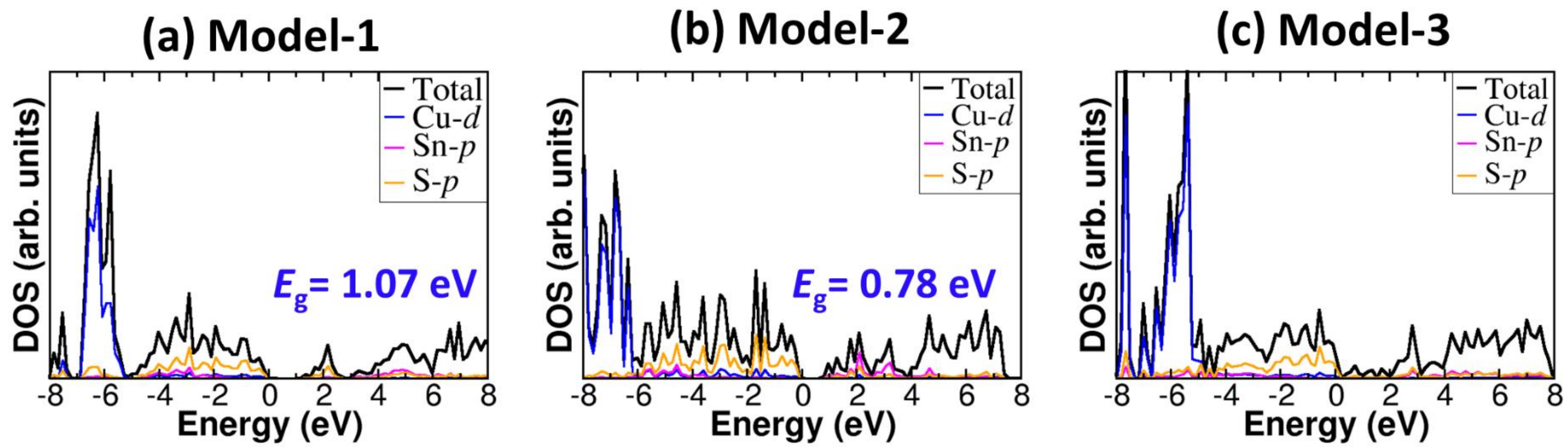

Figure S3: (a) Crystal structure, (b) partial density of states (PDOS), and (c) band structure of ZnS. The calculated corresponding effective masses of holes and electrons along the high-symmetry directions of the Brillouin zone are shown in (d). Atomic color: Zn =grey, and S =yellow.

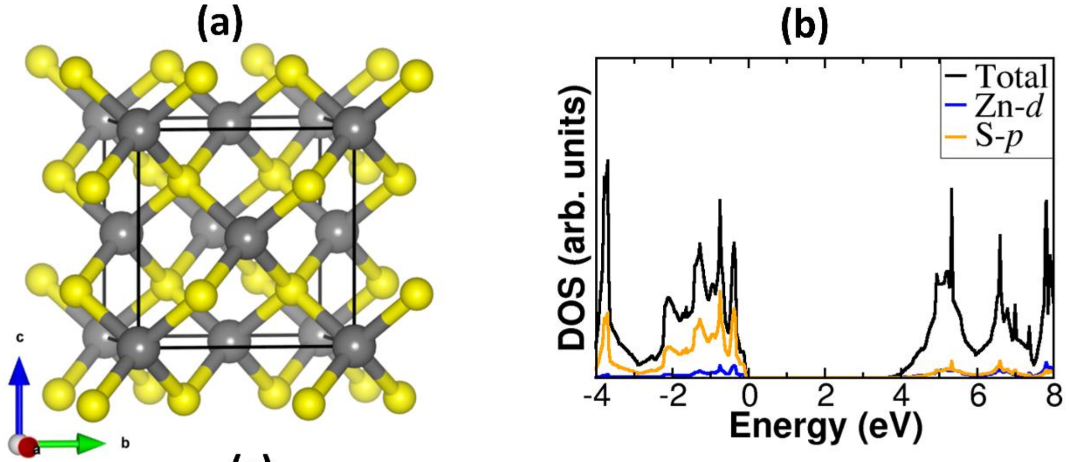

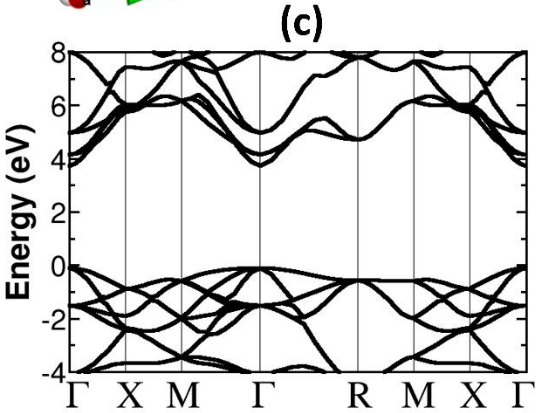

(d)

Direction	m^*_h (m_e)	m^*_e (m_e)
Γ−X	0.0074	0.0097
X−M	0.0099	0.0201
M−Γ	0.0124	0.1028
Γ−R	0.1874	0.0037
R−M	1.1384	0.6086

Figure S4: (a) Crystal structure, (b) partial density of states (PDOS), and (c) band structure of CdS. The calculated corresponding effective masses of holes and electrons along the high-symmetry directions of the Brillouin zone are shown in (d). Atomic color: Cd =black, and S =yellow.

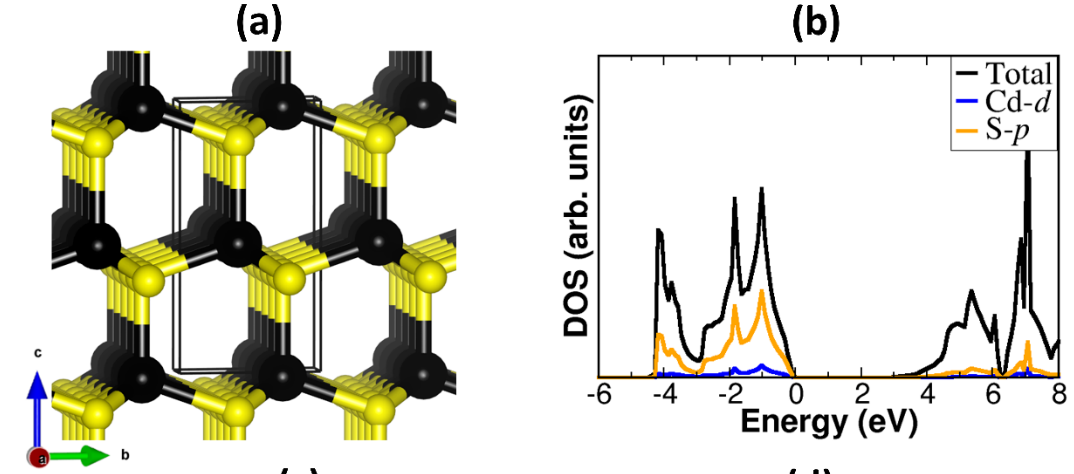

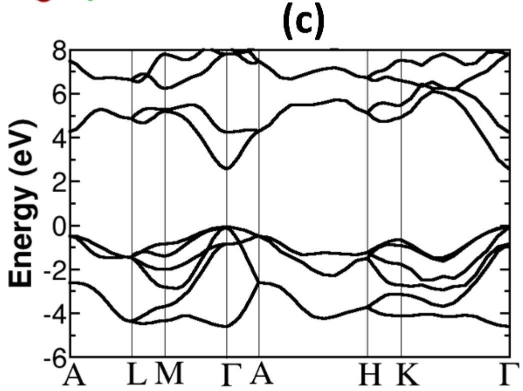

(d)

Direction	m^*_h (m_e)	m^*_e (m_e)
A−L	0.0592	0.0033
L−M	0.0068	0.0025
M−Γ	0.0379	0.0060
Γ−A	0.0088	0.0067
A−H	0.0139	0.0075
H−K	0.0053	0.0025
K−Γ	0.0088	0.0035

Figure S5: Geometry optimized model of the CTS(111) surface used to align the electronic eigenvalues to the vacuum level. The corresponding electrostatic potential is shown in blue.

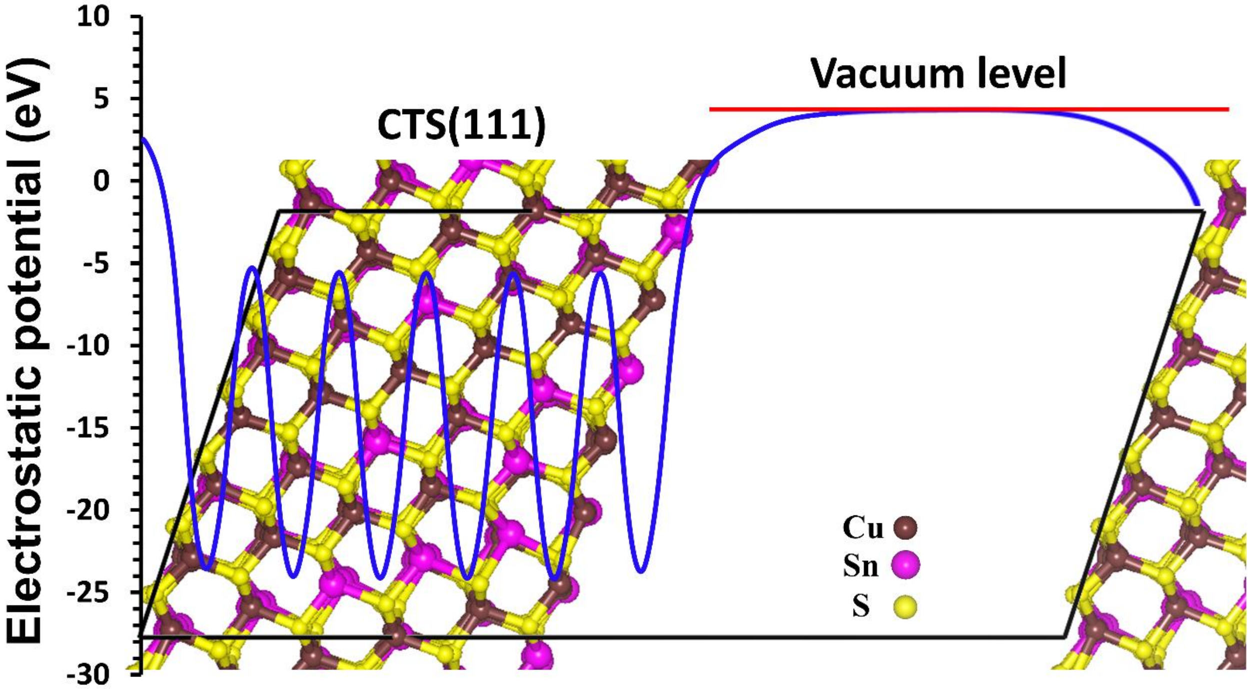

Figure S6: Geometry optimized model of the CdS(100) surface used to align the electronic eigenvalues to the vacuum level. The corresponding electrostatic potential is shown in blue.

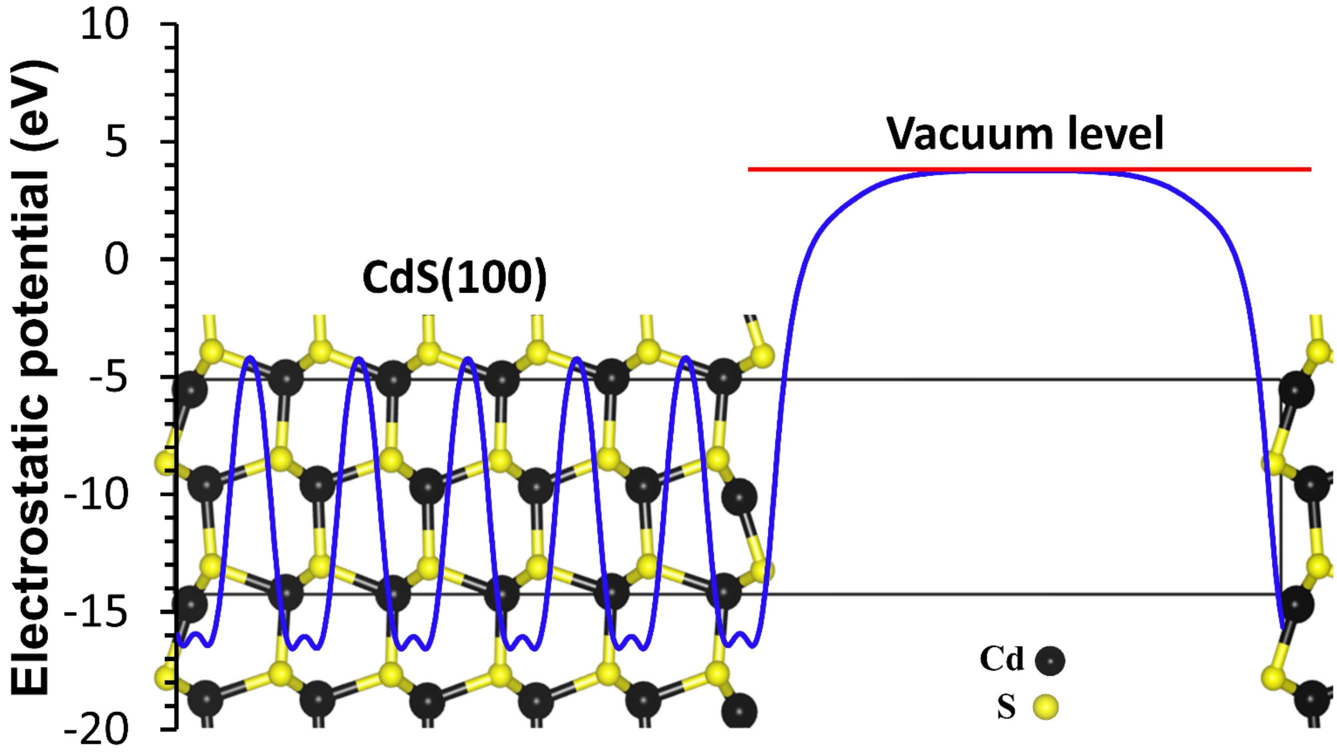

Figure S7: Geometry optimized model of the ZnS(110) surface used to align the electronic eigenvalues to the vacuum level. The corresponding electrostatic potential is shown in blue.

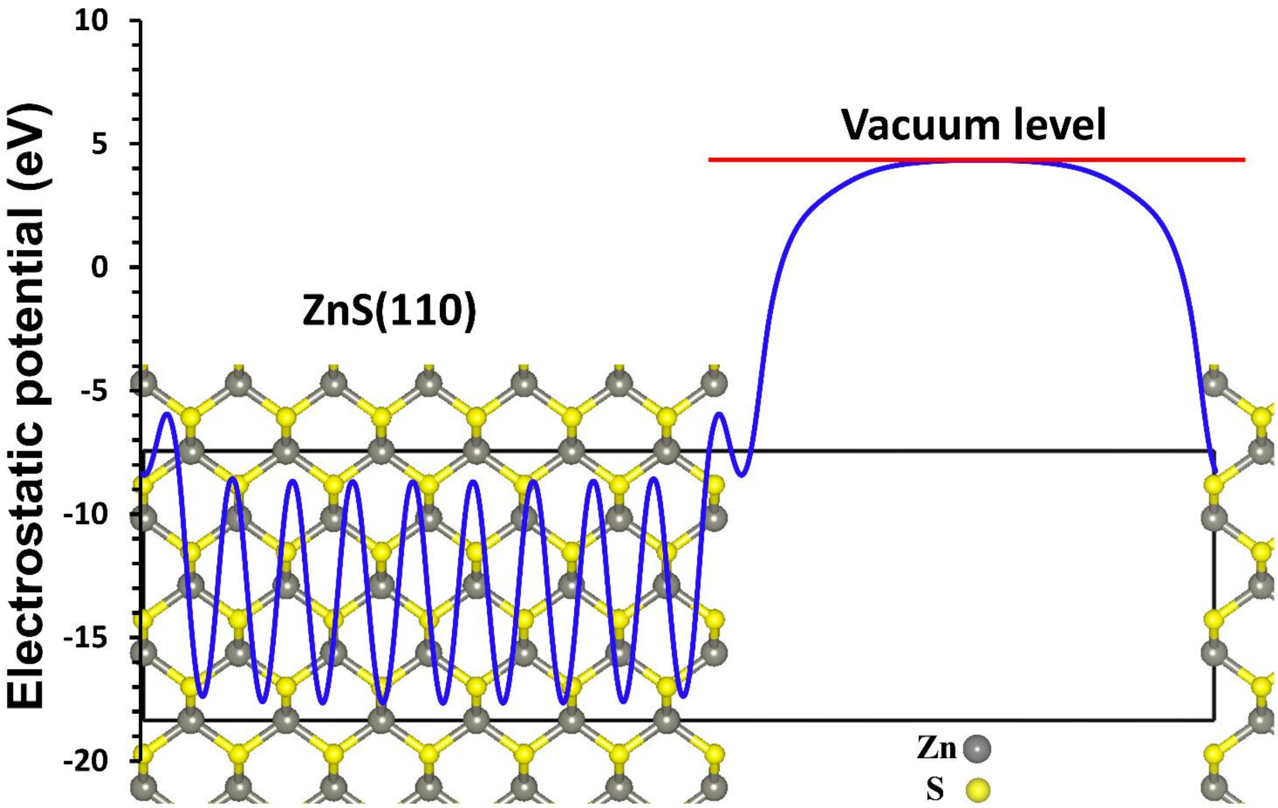

Figure S8: (a) EDX spectra of CTS NPs **(c, d, e)** Elemental mapping of Cu, Sn and S, respectively

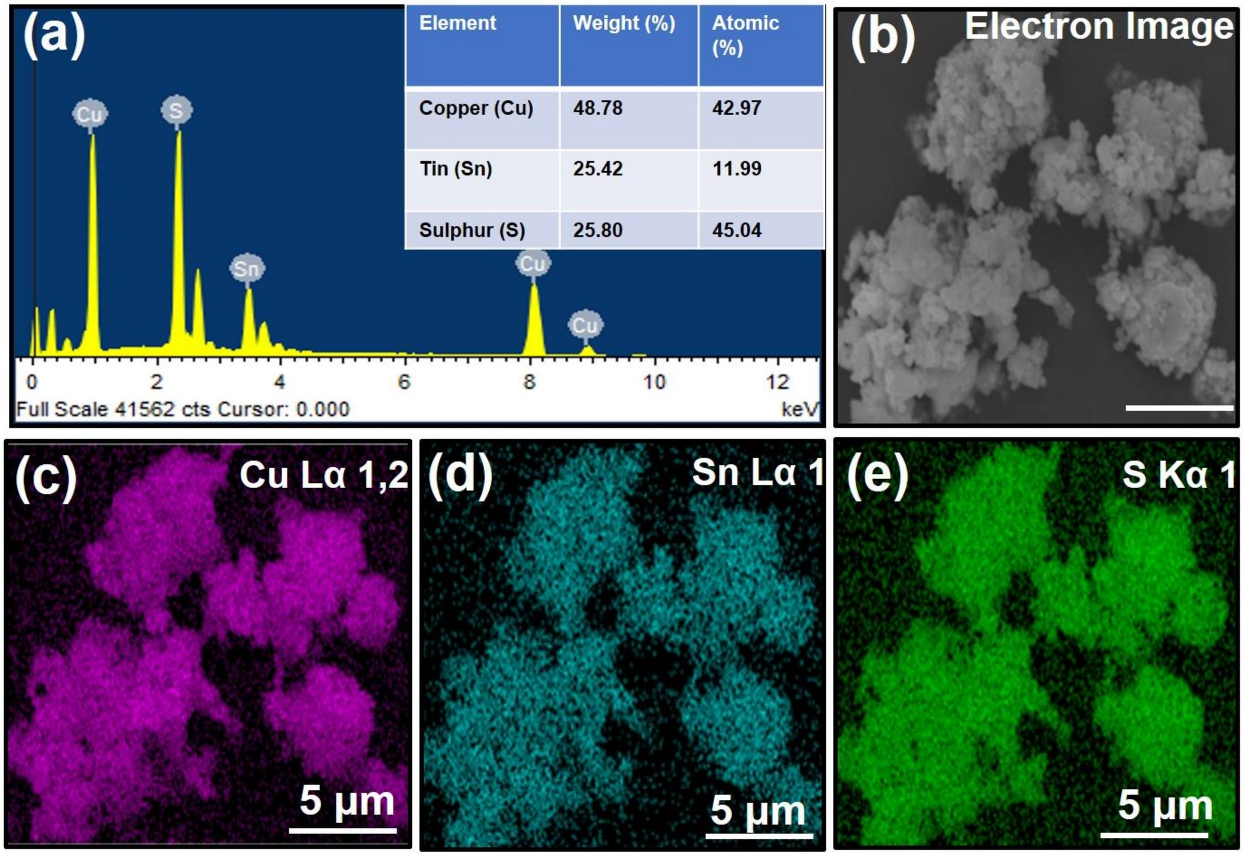

Figure S9: (a, b) SEM images of CTS NPs sample at different magnification

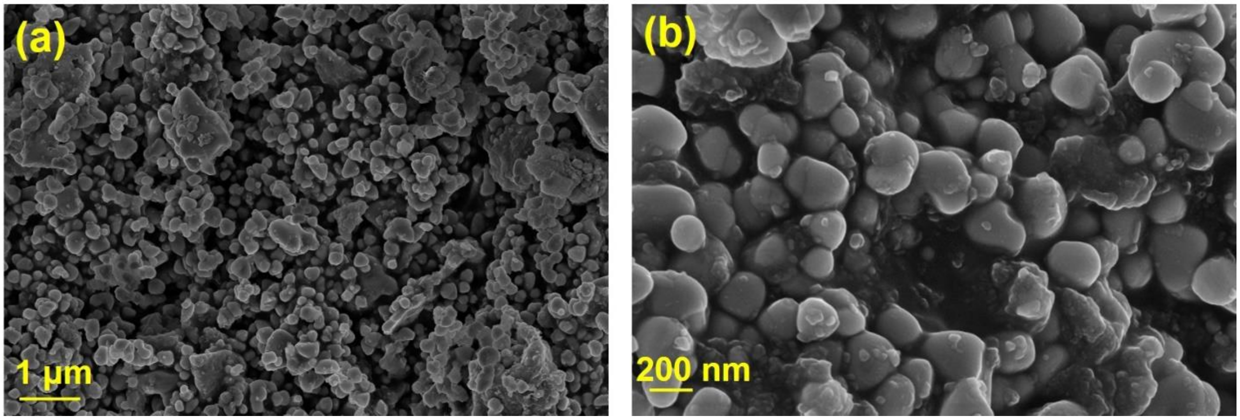

Figure S10: Chronoamperometry photocurrent stability test for the CTS thin film under illumination of the AM1.5G solar light.

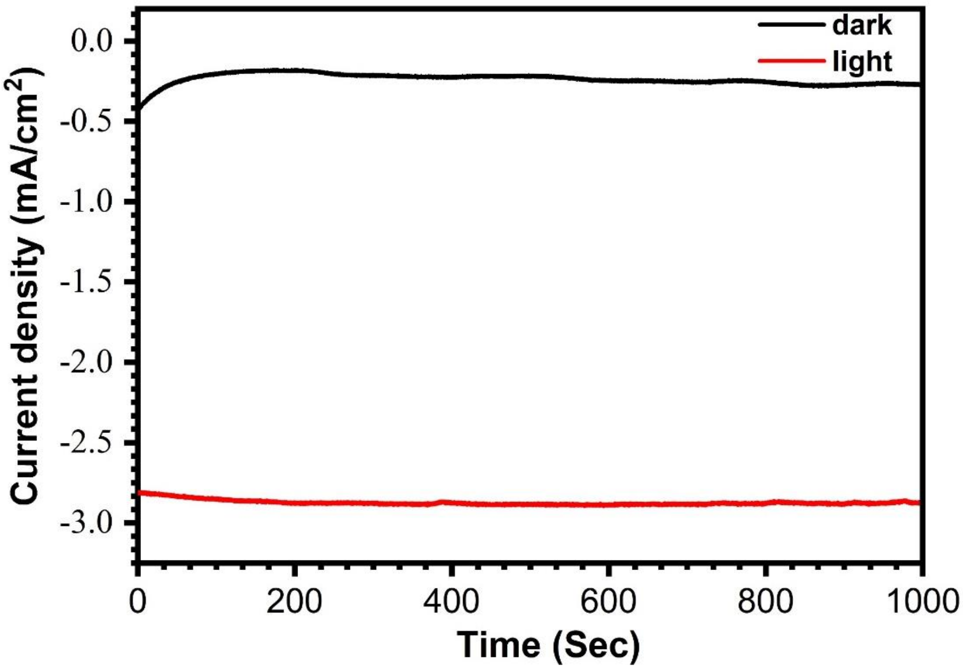

Figure S11: (a) X-ray diffraction pattern of CdS NPs. **(b)** Raman spectrum of CdS NPs. **(c)** UV-visible absorbance spectra of CdS NPs **(d)** band gap (Tauc) plot of CdS NPs.

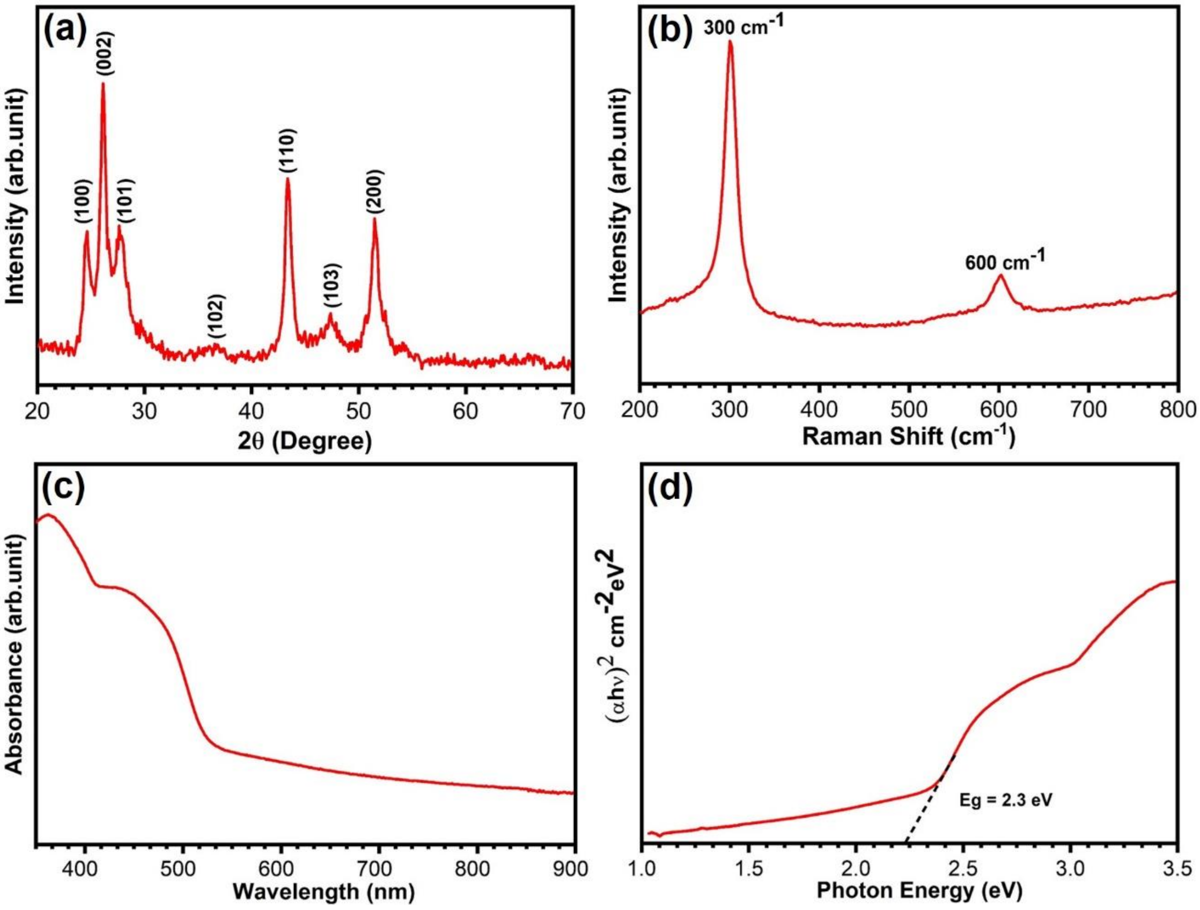

Figure S12: (a) XRD pattern of ZnS NPs synthesized by hot injection method. **(b)** FTIR spectra of ZnS NPs **(c)** UV-vis absorbance spectra of ZnS NPs. **(d)** Band gap plot of ZnS NPs

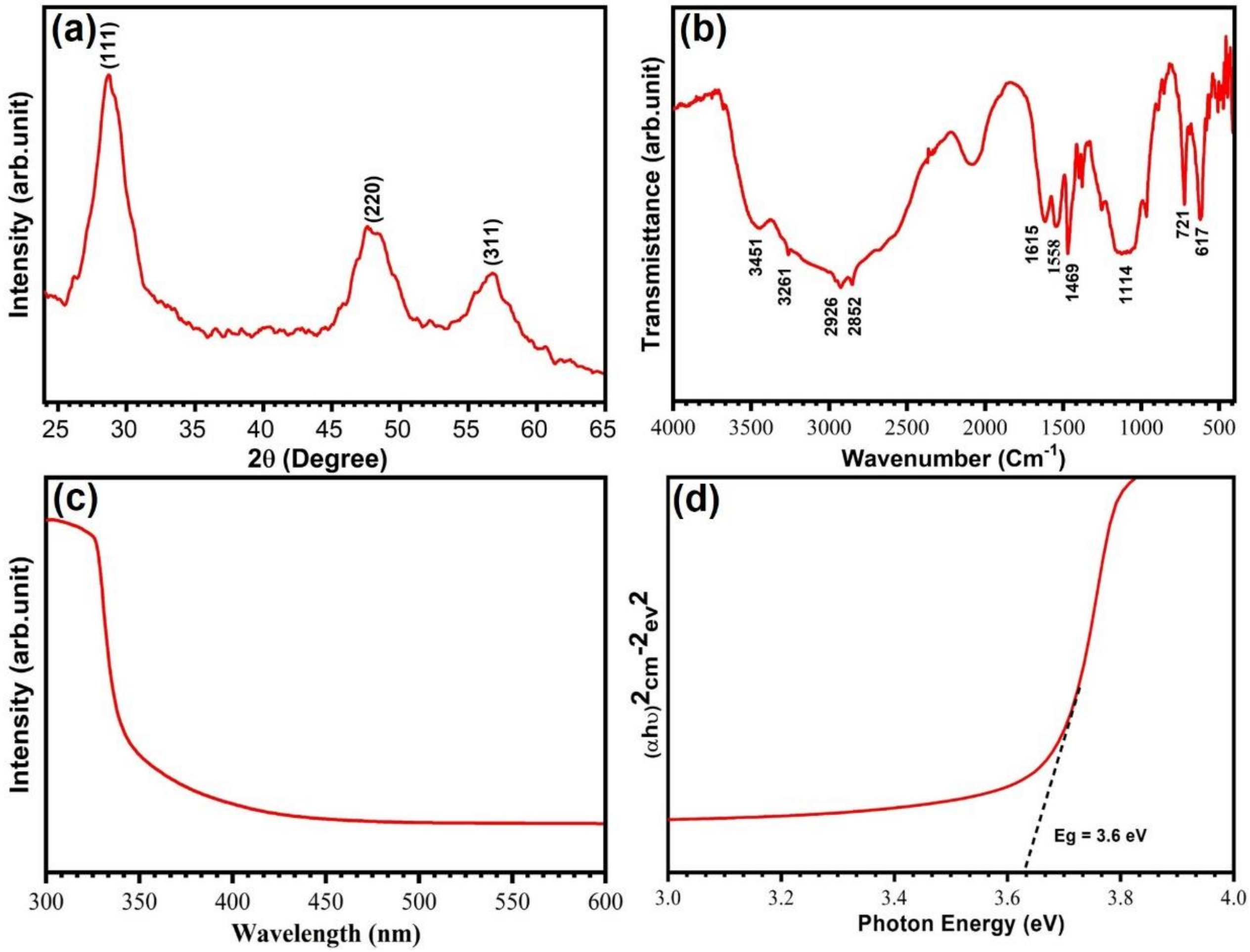

Figure S13: (a) Bode phase plot. **(b)** Z' real vs. -Z" imaginary impedance plot (Nyquist plot) of CdS. **(c)** Mott-Schottky plot. **(d)** Current Density-Potential curves of CdS under simulated AM 1.5 G light irradiation in different conditions.

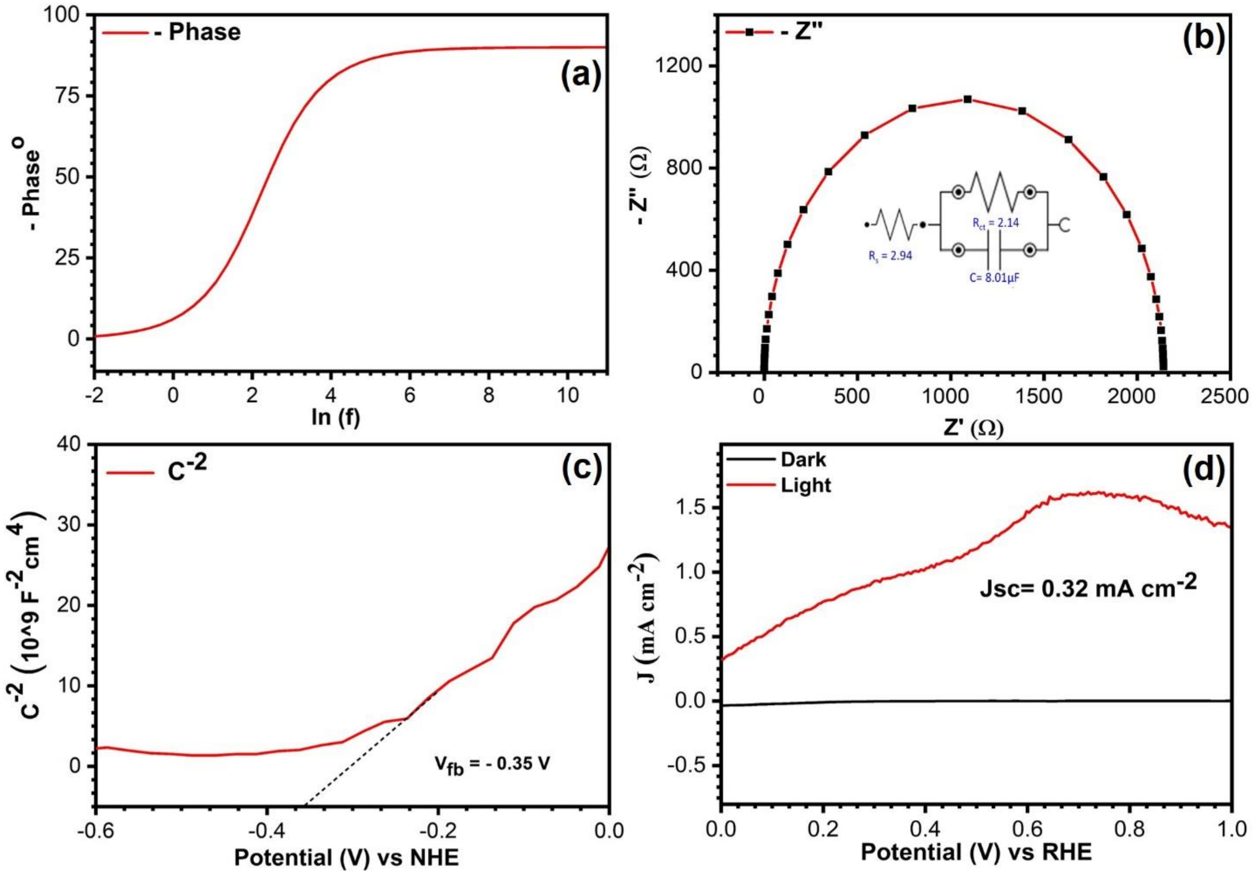

Figure S14: (a) Bode Phase plot. **(b)** Z' real vs. -Z" imaginary impedance plot (Nyquist plot) of ZnS. **(c)** Mott-Schottky plot. **(d)** Current Density-Potential curves of ZnS under simulated AM 1.5 G light irradiation in different conditions.

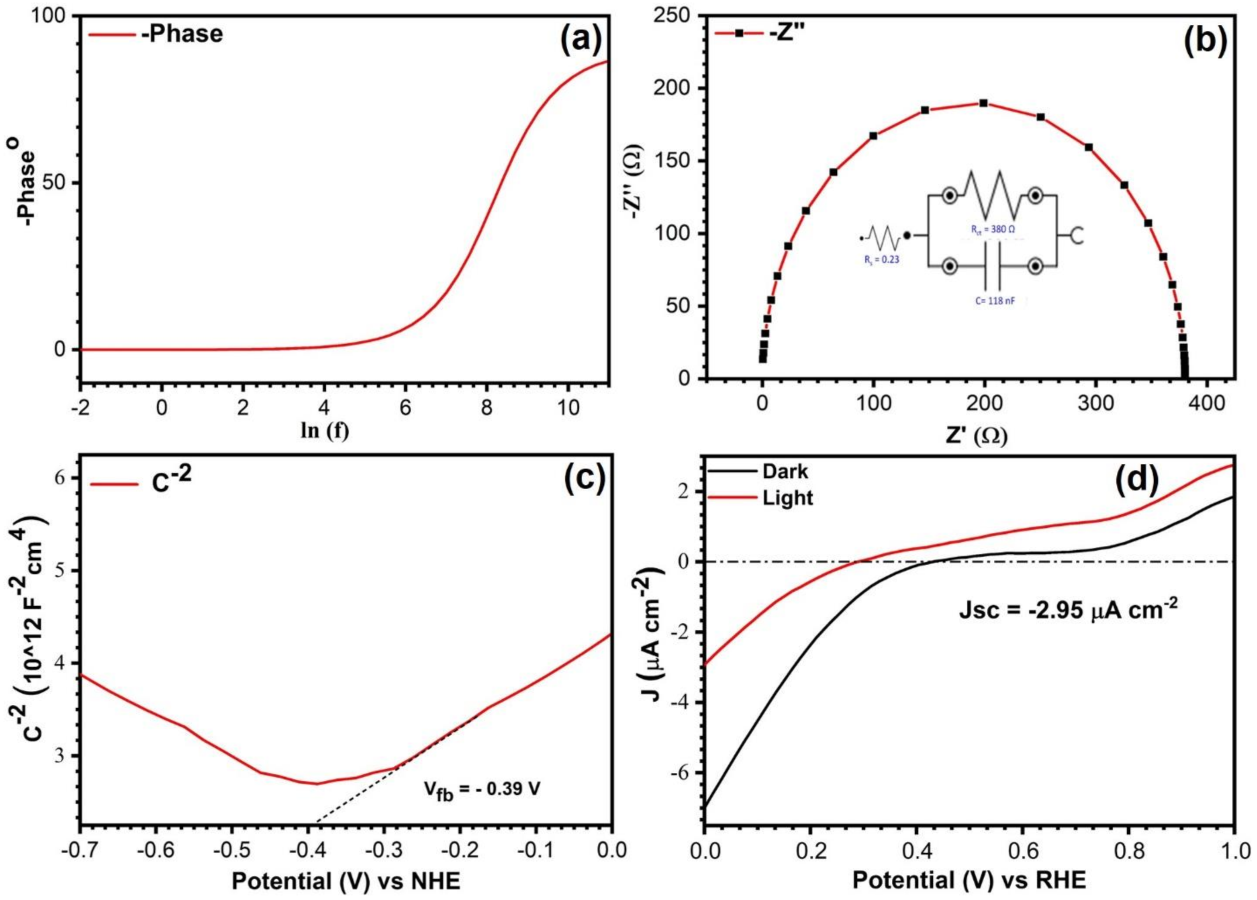

Bulk CTS: Model-1 Structural parameters (VASP)

```
Cu Sn S
   1.00000000000000
    16.2923717227231606    0.0000000000000000    0.0000000000000000
     0.0000000000000000   16.2096464468226920    0.0000000000000000
     0.0000000000000000    0.0000000000000000   16.2869151160225449
    72    36   108
Cartesian
   0.0000000000     0.0000000000     0.0000000000
   0.0000000000     2.7016080000     2.6603860000
   2.7153950000     2.7016080000     0.0000000000
   0.0000000000     2.7016080000     8.1434570000
   2.7153950000     0.0000000000     8.1434570000
   2.7153950000     2.7016080000     5.4830720000
   0.0000000000     2.7016080000    13.6265290000
   2.7153950000     2.7016080000    10.8038430000
   0.0000000000     5.4032150000     0.0000000000
   0.0000000000     8.1048230000     2.6603860000
   2.7153950000     8.1048230000     0.0000000000
   0.0000000000     8.1048230000     8.1434570000
   2.7153950000     5.4032150000     8.1434570000
   2.7153950000     8.1048230000     5.4830720000
   0.0000000000     8.1048230000    13.6265290000
   2.7153950000     8.1048230000    10.8038430000
   0.0000000000    10.8064310000     0.0000000000
   0.0000000000    13.5080390000     2.6603860000
   2.7153950000    13.5080390000     0.0000000000
   0.0000000000    13.5080390000     8.1434570000
   2.7153950000    10.8064310000     8.1434570000
   2.7153950000    13.5080390000     5.4830720000
   0.0000000000    13.5080390000    13.6265290000
   2.7153950000    13.5080390000    10.8038430000
   5.4307910000     0.0000000000     0.0000000000
   5.4307910000     2.7016080000     2.6603860000
   8.1461860000     2.7016080000     0.0000000000
   5.4307910000     2.7016080000     8.1434570000
   8.1461860000     0.0000000000     8.1434570000
   8.1461860000     2.7016080000     5.4830720000
   5.4307910000     2.7016080000    13.6265290000
   8.1461860000     2.7016080000    10.8038430000
   5.4307910000     5.4032150000     0.0000000000
   5.4307910000     8.1048230000     2.6603860000
   8.1461860000     8.1048230000     0.0000000000
   5.4307910000     8.1048230000     8.1434570000
   8.1461860000     5.4032150000     8.1434570000
   8.1461860000     8.1048230000     5.4830720000
   5.4307910000     8.1048230000    13.6265290000
   8.1461860000     8.1048230000    10.8038430000
   5.4307910000    10.8064310000     0.0000000000
   5.4307910000    13.5080390000     2.6603860000
   8.1461860000    13.5080390000     0.0000000000
   5.4307910000    13.5080390000     8.1434570000
   8.1461860000    10.8064310000     8.1434570000
   8.1461860000    13.5080390000     5.4830720000
   5.4307910000    13.5080390000    13.6265290000
   8.1461860000    13.5080390000    10.8038430000
  10.8615820000     0.0000000000     0.0000000000
  10.8615820000     2.7016080000     2.6603860000
  13.5769760000     2.7016080000     0.0000000000
  10.8615820000     2.7016080000     8.1434570000
  13.5769760000     0.0000000000     8.1434570000
```

13.5769760000	2.7016080000	5.4830720000
10.8615820000	2.7016080000	13.6265290000
13.5769760000	2.7016080000	10.8038430000
10.8615820000	5.4032150000	0.0000000000
10.8615820000	8.1048230000	2.6603860000
13.5769760000	8.1048230000	0.0000000000
10.8615820000	8.1048230000	8.1434570000
13.5769760000	5.4032150000	8.1434570000
13.5769760000	8.1048230000	5.4830720000
10.8615820000	8.1048230000	13.6265290000
13.5769760000	8.1048230000	10.8038430000
10.8615820000	10.8064310000	0.0000000000
10.8615820000	13.5080390000	2.6603860000
13.5769760000	13.5080390000	0.0000000000
10.8615820000	13.5080390000	8.1434570000
13.5769760000	10.8064310000	8.1434570000
13.5769760000	13.5080390000	5.4830720000
10.8615820000	13.5080390000	13.6265290000
13.5769760000	13.5080390000	10.8038430000
2.7153950000	0.0000000000	2.5617050000
0.0000000000	0.0000000000	5.5817520000
0.0000000000	0.0000000000	10.7051630000
2.7153950000	0.0000000000	13.7252090000
2.7153950000	5.4032150000	2.5617050000
0.0000000000	5.4032150000	5.5817520000
0.0000000000	5.4032150000	10.7051630000
2.7153950000	5.4032150000	13.7252090000
2.7153950000	10.8064310000	2.5617050000
0.0000000000	10.8064310000	5.5817520000
0.0000000000	10.8064310000	10.7051630000
2.7153950000	10.8064310000	13.7252090000
8.1461860000	0.0000000000	2.5617050000
5.4307910000	0.0000000000	5.5817520000
5.4307910000	0.0000000000	10.7051630000
8.1461860000	0.0000000000	13.7252090000
8.1461860000	5.4032150000	2.5617050000
5.4307910000	5.4032150000	5.5817520000
5.4307910000	5.4032150000	10.7051630000
8.1461860000	5.4032150000	13.7252090000
8.1461860000	10.8064310000	2.5617050000
5.4307910000	10.8064310000	5.5817520000
5.4307910000	10.8064310000	10.7051630000
8.1461860000	10.8064310000	13.7252090000
13.5769760000	0.0000000000	2.5617050000
10.8615820000	0.0000000000	5.5817520000
10.8615820000	0.0000000000	10.7051630000
13.5769760000	0.0000000000	13.7252090000
13.5769760000	5.4032150000	2.5617050000
10.8615820000	5.4032150000	5.5817520000
10.8615820000	5.4032150000	10.7051630000
13.5769760000	5.4032150000	13.7252090000
13.5769760000	10.8064310000	2.5617050000
10.8615820000	10.8064310000	5.5817520000
10.8615820000	10.8064310000	10.7051630000
13.5769760000	10.8064310000	13.7252090000
4.0730930000	1.4940760000	4.0717290000
4.1573790000	3.9626280000	1.2568030000
1.2734120000	1.4405870000	1.2568030000
1.3576980000	3.9091400000	4.0717290000
3.9888070000	1.4405870000	9.4002610000
3.9888070000	3.9626280000	6.8866540000
1.4419840000	1.4405870000	6.8866540000

1.4419840000	3.9626280000	9.4002610000
4.1573790000	1.4405870000	15.0301110000
4.0730930000	3.9091400000	12.2151860000
1.3576980000	1.4940760000	12.2151860000
1.2734120000	3.9626280000	15.0301110000
4.0730930000	6.8972910000	4.0717290000
4.1573790000	9.3658440000	1.2568030000
1.2734120000	6.8438030000	1.2568030000
1.3576980000	9.3123550000	4.0717290000
3.9888070000	6.8438030000	9.4002610000
3.9888070000	9.3658440000	6.8866540000
1.4419840000	6.8438030000	6.8866540000
1.4419840000	9.3658440000	9.4002610000
4.1573790000	6.8438030000	15.0301110000
4.0730930000	9.3123550000	12.2151860000
1.3576980000	6.8972910000	12.2151860000
1.2734120000	9.3658440000	15.0301110000
4.0730930000	12.3005080000	4.0717290000
4.1573790000	14.7690580000	1.2568030000
1.2734120000	12.2470180000	1.2568030000
1.3576980000	14.7155700000	4.0717290000
3.9888070000	12.2470180000	9.4002610000
3.9888070000	14.7690580000	6.8866540000
1.4419840000	12.2470180000	6.8866540000
1.4419840000	14.7690580000	9.4002610000
4.1573790000	12.2470180000	15.0301110000
4.0730930000	14.7155700000	12.2151860000
1.3576980000	12.3005080000	12.2151860000
1.2734120000	14.7690580000	15.0301110000
9.5038830000	1.4940760000	4.0717290000
9.5881700000	3.9626280000	1.2568030000
6.7042020000	1.4405870000	1.2568030000
6.7884880000	3.9091400000	4.0717290000
9.4195980000	1.4405870000	9.4002610000
9.4195980000	3.9626280000	6.8866540000
6.8727740000	1.4405870000	6.8866540000
6.8727740000	3.9626280000	9.4002610000
9.5881700000	1.4405870000	15.0301110000
9.5038830000	3.9091400000	12.2151860000
6.7884880000	1.4940760000	12.2151860000
6.7042020000	3.9626280000	15.0301110000
9.5038830000	6.8972910000	4.0717290000
9.5881700000	9.3658440000	1.2568030000
6.7042020000	6.8438030000	1.2568030000
6.7884880000	9.3123550000	4.0717290000
9.4195980000	6.8438030000	9.4002610000
9.4195980000	9.3658440000	6.8866540000
6.8727740000	6.8438030000	6.8866540000
6.8727740000	9.3658440000	9.4002610000
9.5881700000	6.8438030000	15.0301110000
9.5038830000	9.3123550000	12.2151860000
6.7884880000	6.8972910000	12.2151860000
6.7042020000	9.3658440000	15.0301110000
9.5038830000	12.3005080000	4.0717290000
9.5881700000	14.7690580000	1.2568030000
6.7042020000	12.2470180000	1.2568030000
6.7884880000	14.7155700000	4.0717290000
9.4195980000	12.2470180000	9.4002610000
9.4195980000	14.7690580000	6.8866540000
6.8727740000	12.2470180000	6.8866540000
6.8727740000	14.7690580000	9.4002610000
9.5881700000	12.2470180000	15.0301110000

9.5038830000	14.7155700000	12.2151860000
6.7884880000	12.3005080000	12.2151860000
6.7042020000	14.7690580000	15.0301110000
14.9346740000	1.4940760000	4.0717290000
15.0189600000	3.9626280000	1.2568030000
12.1349930000	1.4405870000	1.2568030000
12.2192780000	3.9091400000	4.0717290000
14.8503880000	1.4405870000	9.4002610000
14.8503880000	3.9626280000	6.8866540000
12.3035650000	1.4405870000	6.8866540000
12.3035650000	3.9626280000	9.4002610000
15.0189600000	1.4405870000	15.0301110000
14.9346740000	3.9091400000	12.2151860000
12.2192780000	1.4940760000	12.2151860000
12.1349930000	3.9626280000	15.0301110000
14.9346740000	6.8972910000	4.0717290000
15.0189600000	9.3658440000	1.2568030000
12.1349930000	6.8438030000	1.2568030000
12.2192780000	9.3123550000	4.0717290000
14.8503880000	6.8438030000	9.4002610000
14.8503880000	9.3658440000	6.8866540000
12.3035650000	6.8438030000	6.8866540000
12.3035650000	9.3658440000	9.4002610000
15.0189600000	6.8438030000	15.0301110000
14.9346740000	9.3123550000	12.2151860000
12.2192780000	6.8972910000	12.2151860000
12.1349930000	9.3658440000	15.0301110000
14.9346740000	12.3005080000	4.0717290000
15.0189600000	14.7690580000	1.2568030000
12.1349930000	12.2470180000	1.2568030000
12.2192780000	14.7155700000	4.0717290000
14.8503880000	12.2470180000	9.4002610000
14.8503880000	14.7690580000	6.8866540000
12.3035650000	12.2470180000	6.8866540000
12.3035650000	14.7690580000	9.4002610000
15.0189600000	12.2470180000	15.0301110000
14.9346740000	14.7155700000	12.2151860000
12.2192780000	12.3005080000	12.2151860000
12.1349930000	14.7690580000	15.0301110000

Bulk CTS: Model-2 Structural parameters (VASP)

```
Cu Sn S
   1.00000000000000
    16.4319356750770709    0.0000000000000000    0.2187760184372871
     0.0000000000000000   16.1000178401744414    0.0000000000000000
     0.2167082976366084    0.0000000000000000   16.3079773495375697
    72    36   108
Cartesian
 0.0000000000    -0.0195080000     0.0000000000
 0.0361180000     2.5366600000     2.7179960000
 2.8353280000    -0.0163350000     2.7022090000
 0.0722360000    -0.0195080000     5.4359930000
 0.1083540000     2.5366600000     8.1539890000
 2.9075640000    -0.0163350000     8.1382020000
 0.1444720000    -0.0195080000    10.8719850000
 0.1805900000     2.5366600000    13.5899810000
 2.9798000000    -0.0163350000    13.5741940000
 0.0000000000     5.2150020000     0.0000000000
 0.0361180000     8.0140840000     2.7179960000
 2.6072700000     5.1748230000     2.5910830000
 0.0722360000     5.2150020000     5.4359930000
 0.1083540000     8.0140840000     8.1539890000
 2.6795060000     5.1748230000     8.0270760000
 0.1444720000     5.2150020000    10.8719850000
 0.1805900000     8.0140840000    13.5899810000
 2.7517420000     5.1748230000    13.4630680000
 0.0000000000    10.8490490000     0.0000000000
 0.0361180000    13.5506680000     2.7179960000
 2.6421840000    10.8914790000     2.7415980000
 0.0722360000    10.8490490000     5.4359930000
 0.1083540000    13.5506680000     8.1539890000
 2.7144200000    10.8914790000     8.1775900000
 0.1444720000    10.8490490000    10.8719850000
 0.1805900000    13.5506680000    13.5899810000
 2.7866560000    10.8914790000    13.6135830000
 5.5086430000    -0.0277960000     0.0158870000
 5.4393830000     2.5955550000     2.6442380000
 8.2520870000    -0.0310420000     2.8273840000
 5.5808790000    -0.0277960000     5.4518800000
 5.5116190000     2.5955550000     8.0802310000
 8.3243230000    -0.0310420000     8.2633770000
 5.6531150000    -0.0277960000    10.8878720000
 5.5838550000     2.5955550000    13.5162230000
 8.3965590000    -0.0310420000    13.6993690000
 5.2528200000     5.1411340000    -0.1896810000
 5.3250570000     5.1411340000     5.2463120000
 5.3972930000     5.1411340000    10.6823040000
 5.3689350000    10.8826540000    -0.0103500000
 5.5202160000    13.4158680000     2.7288640000
 8.2520870000    10.8792140000     2.8273840000
 5.4411710000    10.8826540000     5.4256420000
 5.5924520000    13.4158680000     8.1648560000
 8.3243230000    10.8792140000     8.2633770000
 5.5134070000    10.8826540000    10.8616350000
 5.6646880000    13.4158680000    13.6008490000
 8.3965590000    10.8792140000    13.6993690000
10.9232940000    -0.0277960000     0.2028890000
11.0647900000     2.5955550000     3.0105300000
13.6688440000    -0.0163350000     2.9525590000
10.9955300000    -0.0277960000     5.6388820000
11.1370260000     2.5955550000     8.4465230000
```

13.7410800000	-0.0163350000	8.3885520000
11.0677660000	-0.0277960000	11.0748740000
11.2092620000	2.5955550000	13.8825150000
13.8133160000	-0.0163350000	13.8245450000
11.1791160000	5.1411340000	0.4084570000
13.8969030000	5.1748230000	3.0636850000
11.2513520000	5.1411340000	5.8444500000
13.9691390000	5.1748230000	8.4996790000
11.3235880000	5.1411340000	11.2804410000
14.0413750000	5.1748230000	13.9356710000
11.0630020000	10.8826540000	0.2291260000
10.9839570000	13.4158680000	2.9259050000
13.8619880000	10.8914790000	2.9131710000
11.1352380000	10.8826540000	5.6651190000
11.0561930000	13.4158680000	8.3618970000
13.9342240000	10.8914790000	8.3491630000
11.2074750000	10.8826540000	11.1011100000
11.1284290000	13.4158680000	13.7978900000
14.0064600000	10.8914790000	13.7851550000
5.2237730000	8.0620570000	2.5113880000
8.2520870000	5.7273180000	2.8273840000
5.2960100000	8.0620570000	7.9473810000
8.3243230000	5.7273180000	8.2633770000
5.3682460000	8.0620570000	13.3833730000
8.3965590000	5.7273180000	13.6993690000
11.2803980000	8.0620570000	3.1433810000
11.3526350000	8.0620570000	8.5793730000
11.4248710000	8.0620570000	14.0153660000
2.6949310000	2.5533220000	-0.0375090000
2.7671670000	2.5533220000	5.3984840000
2.8394030000	2.5533220000	10.8344760000
2.4374420000	8.0246690000	-0.0945400000
2.5096780000	8.0246690000	5.3414530000
2.5819140000	8.0246690000	10.7774450000
2.7175010000	13.5201580000	0.0008540000
2.7897370000	13.5201580000	5.4368470000
2.8619740000	13.5201580000	10.8728390000
8.2159680000	2.5658200000	0.1093880000
8.2882040000	2.5658200000	5.5453810000
8.3604400000	2.5658200000	10.9813740000
8.2159680000	8.7691230000	0.1093880000
8.2882040000	8.7691230000	5.5453810000
8.3604400000	8.7691230000	10.9813740000
8.2159680000	13.4851860000	0.1093880000
8.2882040000	13.4851860000	5.5453810000
8.3604400000	13.4851860000	10.9813740000
13.7370060000	2.5533220000	0.2562850000
13.8092420000	2.5533220000	5.6922770000
13.8814780000	2.5533220000	11.1282700000
13.9944950000	8.0246690000	0.3133160000
14.0667310000	8.0246690000	5.7493090000
14.1389680000	8.0246690000	11.1853010000
13.7144350000	13.5201580000	0.2179220000
13.7866710000	13.5201580000	5.6539140000
13.8589070000	13.5201580000	11.0899070000
4.2586620000	1.1969040000	4.0221350000
4.1209430000	3.9760830000	1.3796270000
1.3307850000	1.1649210000	1.4354490000
1.3105850000	3.9523450000	3.9963300000
4.3308980000	1.1969040000	9.4581280000
4.1931790000	3.9760830000	6.8156200000
1.4030210000	1.1649210000	6.8714420000

1.3828210000	3.9523450000	9.4323230000
4.4031340000	1.1969040000	14.8941200000
4.2654150000	3.9760830000	12.2516130000
1.4752570000	1.1649210000	12.3074350000
1.4550570000	3.9523450000	14.8683170000
3.9428830000	6.4633040000	3.9309720000
3.8572190000	9.6421370000	1.2114900000
1.2277220000	6.5567170000	1.4063410000
1.2502590000	9.5332270000	3.9509630000
4.0151190000	6.4633040000	9.3669640000
3.9294550000	9.6421370000	6.6474830000
1.2999580000	6.5567170000	6.8423330000
1.3224960000	9.5332270000	9.3869560000
4.0873550000	6.4633040000	14.8029570000
4.0016910000	9.6421370000	12.0834760000
1.3721950000	6.5567170000	12.2783260000
1.3947320000	9.5332270000	14.8229480000
4.1388070000	12.0896320000	3.9851010000
4.2318680000	14.8107160000	1.4353590000
1.2707630000	12.1303950000	1.4095370000
1.3679410000	14.8907270000	3.9928970000
4.2110440000	12.0896320000	9.4210940000
4.3041040000	14.8107160000	6.8713520000
1.3429990000	12.1303950000	6.8455300000
1.4401770000	14.8907270000	9.4288880000
4.2832800000	12.0896320000	14.8570860000
4.3763400000	14.8107160000	12.3073440000
1.4152360000	12.1303950000	12.2815220000
1.5124140000	14.8907270000	14.8648820000
9.7029320000	1.1693250000	4.1589020000
9.7195820000	3.9082810000	1.6215990000
6.8012390000	1.1693250000	1.4958670000
6.7845910000	3.9082810000	4.0331700000
9.7751680000	1.1693250000	9.5948930000
9.7918180000	3.9082810000	7.0575920000
6.8734750000	1.1693250000	6.9318590000
6.8568270000	3.9082810000	9.4691630000
9.8474040000	1.1693250000	15.0308870000
9.8640540000	3.9082810000	12.4935840000
6.9457110000	1.1693250000	12.3678520000
6.9290630000	3.9082810000	14.9051550000
10.0298190000	6.7727580000	4.8043910000
9.9855120000	9.6494370000	1.8942690000
6.4743530000	6.7727580000	0.8503780000
6.5186610000	9.6494370000	3.7604990000
10.1020560000	6.7727580000	10.2403840000
10.0577480000	9.6494370000	7.3302620000
6.5465890000	6.7727580000	6.2863710000
6.5908980000	9.6494370000	9.1964910000
10.1742920000	6.7727580000	15.6763760000
10.1299840000	9.6494370000	12.7662540000
6.6188250000	6.7727580000	11.7223630000
6.6631340000	9.6494370000	14.6324830000
9.7107930000	11.9830710000	4.2352510000
9.6745920000	14.7719360000	1.6059280000
6.7933800000	11.9830710000	1.4195170000
6.8295800000	14.7719360000	4.0488410000
9.7830290000	11.9830710000	9.6712440000
9.7468280000	14.7719360000	7.0419210000
6.8656160000	11.9830710000	6.8555100000
6.9018160000	14.7719360000	9.4848330000
9.8552650000	11.9830710000	15.1072360000

9.8190640000	14.7719360000	12.4779130000
6.9378520000	11.9830710000	12.2915020000
6.9740520000	14.7719360000	14.9208250000
15.1733880000	1.1649210000	4.2193190000
15.1935880000	3.9523450000	1.6584380000
12.2455100000	1.1969040000	1.6326340000
12.3832300000	3.9760830000	4.2751420000
15.2456240000	1.1649210000	9.6553110000
15.2658240000	3.9523450000	7.0944310000
12.3177460000	1.1969040000	7.0686260000
12.4554660000	3.9760830000	9.7111340000
15.3178600000	1.1649210000	15.0913040000
15.3380600000	3.9523450000	12.5304230000
12.3899820000	1.1969040000	12.5046200000
12.5277020000	3.9760830000	15.1471260000
15.2764490000	6.5567170000	4.2484270000
15.2539130000	9.5332270000	1.7038050000
12.5612900000	6.4633040000	1.7237970000
12.6469530000	9.6421370000	4.4432780000
15.3486850000	6.5567170000	9.6844200000
15.3261490000	9.5332270000	7.1397980000
12.6335260000	6.4633040000	7.1597900000
12.7191890000	9.6421370000	9.8792710000
15.4209220000	6.5567170000	15.1204130000
15.3983850000	9.5332270000	12.5757900000
12.7057620000	6.4633040000	12.5957820000
12.7914250000	9.6421370000	15.3152640000
15.2334090000	12.1303950000	4.2452310000
15.1362300000	14.8907270000	1.6618720000
12.3653650000	12.0896320000	1.6696670000
12.2723040000	14.8107160000	4.2194090000
15.3056450000	12.1303950000	9.6812240000
15.2084670000	14.8907270000	7.0978640000
12.4376010000	12.0896320000	7.1056600000
12.3445400000	14.8107160000	9.6554010000
15.3778810000	12.1303950000	15.1172160000
15.2807030000	14.8907270000	12.5338570000
12.5098370000	12.0896320000	12.5416530000
12.4167770000	14.8107160000	15.0913940000

Bulk CTS: Model-3 Structural parameters (VASP)

```
Cu Sn S
   1.00000000000000
    16.4148186127710858   -0.0000000000000000   -0.0000000000000000
     0.0000000000000000   16.4148186127710858   -0.0000000000000000
     0.0000000000000000   -0.0000000000000000   16.3814274087187606
    72    36   108
Cartesian
  0.0000000000     0.0000000000     0.0000000000
  0.0000000000     2.7358030000     2.3552690000
  2.7358030000     0.0000000000     2.3552690000
  0.0000000000     2.7358030000     8.1907140000
  2.7358030000     0.0000000000     8.1907140000
  0.0000000000     2.7358030000    14.0261590000
  2.7358030000     0.0000000000    14.0261590000
  0.0000000000     5.4716060000     0.0000000000
  0.0000000000     8.2074090000     2.3552690000
  2.7358030000     5.4716060000     2.3552690000
  0.0000000000     8.2074090000     8.1907140000
  2.7358030000     5.4716060000     8.1907140000
  0.0000000000     8.2074090000    14.0261590000
  2.7358030000     5.4716060000    14.0261590000
  0.0000000000    10.9432130000     0.0000000000
  0.0000000000    13.6790140000     2.3552690000
  2.7358030000    10.9432130000     2.3552690000
  0.0000000000    13.6790140000     8.1907140000
  2.7358030000    10.9432130000     8.1907140000
  0.0000000000    13.6790140000    14.0261590000
  2.7358030000    10.9432130000    14.0261590000
  5.4716060000     0.0000000000     0.0000000000
  5.4716060000     2.7358030000     2.3552690000
  8.2074090000     0.0000000000     2.3552690000
  5.4716060000     2.7358030000     8.1907140000
  8.2074090000     0.0000000000     8.1907140000
  5.4716060000     2.7358030000    14.0261590000
  8.2074090000     0.0000000000    14.0261590000
  5.4716060000     5.4716060000     0.0000000000
  5.4716060000    10.9432130000     0.0000000000
  5.4716060000    13.6790140000     2.3552690000
  8.2074090000    10.9432130000     2.3552690000
  5.4716060000    13.6790140000     8.1907140000
  8.2074090000    10.9432130000     8.1907140000
  5.4716060000    13.6790140000    14.0261590000
  8.2074090000    10.9432130000    14.0261590000
 10.9432130000     0.0000000000     0.0000000000
 10.9432130000     2.7358030000     2.3552690000
 13.6790140000     0.0000000000     2.3552690000
 10.9432130000     2.7358030000     8.1907140000
 13.6790140000     0.0000000000     8.1907140000
 10.9432130000     2.7358030000    14.0261590000
 13.6790140000     0.0000000000    14.0261590000
 10.9432130000     5.4716060000     0.0000000000
 13.6790140000     5.4716060000     2.3552690000
 13.6790140000     5.4716060000     8.1907140000
 13.6790140000     5.4716060000    14.0261590000
 10.9432130000    10.9432130000     0.0000000000
 10.9432130000    13.6790140000     2.3552690000
 13.6790140000    10.9432130000     2.3552690000
 10.9432130000    13.6790140000     8.1907140000
 13.6790140000    10.9432130000     8.1907140000
 10.9432130000    13.6790140000    14.0261590000
```

13.6790140000	10.9432130000	14.0261590000
5.4716060000	8.2074090000	2.3552690000
8.2074090000	5.4716060000	2.3552690000
5.4716060000	8.2074090000	8.1907140000
8.2074090000	5.4716060000	8.1907140000
5.4716060000	8.2074090000	14.0261590000
8.2074090000	5.4716060000	14.0261590000
10.9432130000	8.2074090000	2.3552690000
10.9432130000	8.2074090000	8.1907140000
10.9432130000	8.2074090000	14.0261590000
2.7358030000	2.7358030000	0.0000000000
2.7358030000	8.2074090000	0.0000000000
2.7358030000	13.6790140000	0.0000000000
8.2074090000	2.7358030000	0.0000000000
8.2074090000	8.2074090000	0.0000000000
8.2074090000	13.6790140000	0.0000000000
13.6790140000	2.7358030000	0.0000000000
13.6790140000	8.2074090000	0.0000000000
13.6790140000	13.6790140000	0.0000000000
0.0000000000	0.0000000000	5.2671290000
0.0000000000	5.4716060000	5.2671290000
0.0000000000	10.9432130000	5.2671290000
5.4716060000	0.0000000000	5.2671290000
5.4716060000	5.4716060000	5.2671290000
5.4716060000	10.9432130000	5.2671290000
10.9432130000	0.0000000000	5.2671290000
10.9432130000	5.4716060000	5.2671290000
10.9432130000	10.9432130000	5.2671290000
2.7358030000	2.7358030000	5.2671290000
2.7358030000	8.2074090000	5.2671290000
2.7358030000	13.6790140000	5.2671290000
8.2074090000	2.7358030000	5.2671290000
8.2074090000	8.2074090000	5.2671290000
8.2074090000	13.6790140000	5.2671290000
13.6790140000	2.7358030000	5.2671290000
13.6790140000	8.2074090000	5.2671290000
13.6790140000	13.6790140000	5.2671290000
0.0000000000	0.0000000000	11.1142990000
0.0000000000	5.4716060000	11.1142990000
0.0000000000	10.9432130000	11.1142990000
5.4716060000	0.0000000000	11.1142990000
5.4716060000	5.4716060000	11.1142990000
5.4716060000	10.9432130000	11.1142990000
10.9432130000	0.0000000000	11.1142990000
10.9432130000	5.4716060000	11.1142990000
10.9432130000	10.9432130000	11.1142990000
2.7358030000	2.7358030000	11.1142990000
2.7358030000	8.2074090000	11.1142990000
2.7358030000	13.6790140000	11.1142990000
8.2074090000	2.7358030000	11.1142990000
8.2074090000	8.2074090000	11.1142990000
8.2074090000	13.6790140000	11.1142990000
13.6790140000	2.7358030000	11.1142990000
13.6790140000	8.2074090000	11.1142990000
13.6790140000	13.6790140000	11.1142990000
4.1037040000	1.3679020000	3.6009640000
4.1037040000	4.1037040000	1.2063430000
1.3679020000	1.3679020000	1.2063430000
1.3679020000	4.1037040000	3.6009640000
4.1037040000	1.3679020000	9.3997130000
4.1037040000	4.1037040000	6.9817160000
1.3679020000	1.3679020000	6.9817160000

1.3679020000	4.1037040000	9.3997130000
4.1037040000	1.3679020000	15.1750850000
4.1037040000	4.1037040000	12.7804640000
1.3679020000	1.3679020000	12.7804640000
1.3679020000	4.1037040000	15.1750850000
4.1037040000	6.8395070000	3.6009640000
4.1037040000	9.5753100000	1.2063430000
1.3679020000	6.8395070000	1.2063430000
1.3679020000	9.5753100000	3.6009640000
4.1037040000	6.8395070000	9.3997130000
4.1037040000	9.5753100000	6.9817160000
1.3679020000	6.8395070000	6.9817160000
1.3679020000	9.5753100000	9.3997130000
4.1037040000	6.8395070000	15.1750850000
4.1037040000	9.5753100000	12.7804640000
1.3679020000	6.8395070000	12.7804640000
1.3679020000	9.5753100000	15.1750850000
4.1037040000	12.3111130000	3.6009640000
4.1037040000	15.0469170000	1.2063430000
1.3679020000	12.3111130000	1.2063430000
1.3679020000	15.0469170000	3.6009640000
4.1037040000	12.3111130000	9.3997130000
4.1037040000	15.0469170000	6.9817160000
1.3679020000	12.3111130000	6.9817160000
1.3679020000	15.0469170000	9.3997130000
4.1037040000	12.3111130000	15.1750850000
4.1037040000	15.0469170000	12.7804640000
1.3679020000	12.3111130000	12.7804640000
1.3679020000	15.0469170000	15.1750850000
9.5753100000	1.3679020000	3.6009640000
9.5753100000	4.1037040000	1.2063430000
6.8395070000	1.3679020000	1.2063430000
6.8395070000	4.1037040000	3.6009640000
9.5753100000	1.3679020000	9.3997130000
9.5753100000	4.1037040000	6.9817160000
6.8395070000	1.3679020000	6.9817160000
6.8395070000	4.1037040000	9.3997130000
9.5753100000	1.3679020000	15.1750850000
9.5753100000	4.1037040000	12.7804640000
6.8395070000	1.3679020000	12.7804640000
6.8395070000	4.1037040000	15.1750850000
9.5753100000	6.8395070000	3.6009640000
9.5753100000	9.5753100000	1.2063430000
6.8395070000	6.8395070000	1.2063430000
6.8395070000	9.5753100000	3.6009640000
9.5753100000	6.8395070000	9.3997130000
9.5753100000	9.5753100000	6.9817160000
6.8395070000	6.8395070000	6.9817160000
6.8395070000	9.5753100000	9.3997130000
9.5753100000	6.8395070000	15.1750850000
9.5753100000	9.5753100000	12.7804640000
6.8395070000	6.8395070000	12.7804640000
6.8395070000	9.5753100000	15.1750850000
9.5753100000	12.3111130000	3.6009640000
9.5753100000	15.0469170000	1.2063430000
6.8395070000	12.3111130000	1.2063430000
6.8395070000	15.0469170000	3.6009640000
9.5753100000	12.3111130000	9.3997130000
9.5753100000	15.0469170000	6.9817160000
6.8395070000	12.3111130000	6.9817160000
6.8395070000	15.0469170000	9.3997130000
9.5753100000	12.3111130000	15.1750850000

9.5753100000	15.0469170000	12.7804640000
6.8395070000	12.3111130000	12.7804640000
6.8395070000	15.0469170000	15.1750850000
15.0469170000	1.3679020000	3.6009640000
15.0469170000	4.1037040000	1.2063430000
12.3111130000	1.3679020000	1.2063430000
12.3111130000	4.1037040000	3.6009640000
15.0469170000	1.3679020000	9.3997130000
15.0469170000	4.1037040000	6.9817160000
12.3111130000	1.3679020000	6.9817160000
12.3111130000	4.1037040000	9.3997130000
15.0469170000	1.3679020000	15.1750850000
15.0469170000	4.1037040000	12.7804640000
12.3111130000	1.3679020000	12.7804640000
12.3111130000	4.1037040000	15.1750850000
15.0469170000	6.8395070000	3.6009640000
15.0469170000	9.5753100000	1.2063430000
12.3111130000	6.8395070000	1.2063430000
12.3111130000	9.5753100000	3.6009640000
15.0469170000	6.8395070000	9.3997130000
15.0469170000	9.5753100000	6.9817160000
12.3111130000	6.8395070000	6.9817160000
12.3111130000	9.5753100000	9.3997130000
15.0469170000	6.8395070000	15.1750850000
15.0469170000	9.5753100000	12.7804640000
12.3111130000	6.8395070000	12.7804640000
12.3111130000	9.5753100000	15.1750850000
15.0469170000	12.3111130000	3.6009640000
15.0469170000	15.0469170000	1.2063430000
12.3111130000	12.3111130000	1.2063430000
12.3111130000	15.0469170000	3.6009640000
15.0469170000	12.3111130000	9.3997130000
15.0469170000	15.0469170000	6.9817160000
12.3111130000	12.3111130000	6.9817160000
12.3111130000	15.0469170000	9.3997130000
15.0469170000	12.3111130000	15.1750850000
15.0469170000	15.0469170000	12.7804640000
12.3111130000	12.3111130000	12.7804640000
12.3111130000	15.0469170000	15.1750850000

Bulk CdS: Structural parameters (VASP)

```
Cd S
   1.0000000000000000
     4.1560896338577944    0.0000000069236663   -0.0000000000000000
    -2.0780453049367553    3.5992790341404586    0.0000000000000000
     0.0000000000000000    0.0000000000000000    6.7600040349108603
     2     2
Cartesian
-0.0000030000     2.3995200000     0.0012320000
 2.0780470000     1.1997580000     3.3812340000
-0.0000030000     2.3995200000     2.5472890000
 2.0780470000     1.1997580000     5.9272910000
```

Bulk ZnS: Structural parameters (VASP)

```
Zn S
   1.0000000000000000
     5.4074360910337922   -0.0000000000000000   -0.0000000000000000
     0.0000000000000000    5.4074360910337922    0.0000000000000000
    -0.0000000000000000    0.0000000000000000    5.4074360910337922
     4     4
Cartesian
 0.0000000000     0.0000000000     0.0000000000
 0.0000000000     2.6937180000     2.6937180000
 2.6937180000     0.0000000000     2.6937180000
 2.6937180000     2.6937180000     0.0000000000
 4.0405770000     1.3468590000     4.0405770000
 4.0405770000     4.0405770000     1.3468590000
 1.3468590000     1.3468590000     1.3468590000
 1.3468590000     4.0405770000     4.0405770000
```

CTS (111) surface: Structural parameters (VASP)

```
Cu  Sn  S
1.0
      22.8522052765         0.0000000000         0.0000000000
      11.3051514310        19.8729397395         0.0000000000
       0.0000000000       -25.8086517789        38.9657881411
44   71  216
Cartesian
     7.430366805         4.969325343         0.718451211
    11.273397631         4.999465712         0.834997891
     9.292078087         8.274316421         0.961091161
    15.073543667         5.111150950         1.050127985
    16.957732833         8.421411121         1.051530727
    13.057825808        14.904984095         1.108498762
    11.159062417        11.623809908         1.112629113
    15.007279368        18.037810520         1.117655674
    13.189352459         1.702401230         1.121123665
    22.731990957        11.623459911         1.124318830
     7.415823120        11.631846981         1.126267152
    18.841931031        11.630369819         1.127358166
    14.951845703        11.631237666         1.134839582
    26.524794610        11.642455464         1.138034819
    18.882624491        18.050838690         1.152023562
    28.394387077        14.949333380         1.168817790
    22.610285953        18.138287868         1.212888096
    20.726104833        14.818023290         1.218849844
    24.623383759         8.463777823         1.218927794
    18.873661207         5.283077273         1.220992969
    11.184197520        18.126987688         1.221187773
    22.716706093         5.385340185         1.284546137
    26.337985082        18.170258435         1.314549895
    30.213325902        18.199177593         1.337227920
    11.198747123         7.292178820         3.879433867
    15.041777272         7.322318645         3.995980475
    13.060458069        10.597195025         4.122034988
    18.841922964         7.434004475         4.211110569
    20.726124947        10.744310502         4.212474482
     5.521054548        -2.645076563         4.269442371
    14.927442398        13.946662696         4.273611915
     7.470531074         0.487723569         4.278638476
    16.957732865         4.025280030         4.282067419
    26.500371280        13.946338515         4.285262656
    11.184215578        13.954719955         4.287249881
    22.610311353        13.953222656         4.288340895
    18.720236800        13.954110592         4.295822384
    30.293174932        13.965334212         4.298978428
    11.345853213         0.500751491         4.313006291
    20.857616126        -2.600727334         4.329761399
    15.073526468         0.588221398         4.373870826
    13.189333577        -2.732063824         4.379832500
    28.391775536        10.786676708         4.379871403
    22.642052647         7.605975469         4.381936723
     3.647436503         0.576921267         4.382170429
    26.485086415         7.708193070         4.445528793
    18.801214142         0.620197644         4.475493649
```

22.676554625	0.649090346	4.498210722
14.967138223	9.615076920	7.040377767
18.810157594	9.645197393	7.156924083
16.828838724	12.920048998	7.283017790
22.610303286	9.756883223	7.372054178
24.494505269	13.067163483	7.373456993
9.289445964	-0.322203483	7.430424883
7.390682320	-3.603378307	7.434555524
11.238910715	2.810602317	7.439582085
20.726111825	6.348132819	7.443050221
18.963611626	-3.603728299	7.446245458
3.647444466	-3.595340444	7.448193490
15.073540413	-3.596838040	7.449284504
11.183465522	-3.595976463	7.456804895
22.756404160	-3.584752146	7.459961230
15.114233023	2.823630535	7.473949900
24.626007914	-0.277854390	7.490744201
18.841930114	2.911100442	7.534814434
16.957714046	-0.409185009	7.540776400
32.160155858	13.109529497	7.540854205
26.410432281	9.928829443	7.542919525
7.415816823	2.899800311	7.543114038
30.253465375	10.031071818	7.606472402
22.569594632	2.943050921	7.636476161
26.444935116	2.971969583	7.659154041
18.735518546	11.937929902	10.201360278
22.578549372	11.968070511	10.317906595
9.292078734	-4.629992449	10.443961399
26.378695063	12.079756341	10.533036689
16.957734160	-4.482897893	10.534401183
13.057826455	2.000675561	10.591368492
11.159062726	-1.280525562	10.595538616
15.007302493	5.133475435	10.600564597
24.494503603	8.671031703	10.603993830
22.731991948	-1.280849166	10.607188486
7.415824447	-1.272487848	10.609176582
18.841932022	-1.273965218	10.610267016
14.951845332	-1.273097804	10.617749085
3.672589299	-1.261853646	10.620905419
18.882624800	5.146503653	10.634932411
5.542183128	2.044998887	10.651726712
22.610287618	5.233954015	10.695796946
20.726105824	1.913688109	10.701758911
1.771179473	-4.440531880	10.701798394
30.178812604	12.251708190	10.703863134
11.184197142	5.222653884	10.704096550
11.169651876	12.353944551	10.767455494
26.337986073	5.265949213	10.797419769
7.361121617	5.294842701	10.820136552
11.198747093	-5.612130498	13.362303887
15.041777069	-5.581990185	13.478850204
13.060458375	-2.307139083	13.604943329
18.841923954	-5.470304651	13.693980298
20.726113121	-2.160044528	13.695383113
16.826206433	4.323528750	13.752351584
14.927442367	1.042353186	13.756482225

18.775682815	7.456354183	13.761508206
16.957732673	-8.879055125	13.764976922
26.500370908	1.042003430	13.768171578
11.184204766	1.050391492	13.770120191
22.610312344	1.048887378	13.771250108
18.720225654	1.049755562	13.778731015
7.440970469	1.061000312	13.781887350
22.651005123	7.469382401	13.795876020
9.310562432	4.367877042	13.812670321
26.378700515	7.556851716	13.856740555
24.494484784	4.236540705	13.862742003
5.539559285	-2.117678514	13.862780325
22.642052613	-5.298359408	13.864846226
14.952577124	7.545506480	13.865079642
3.632881189	-5.196115848	13.928399103
30.106365033	7.588801810	13.958402862
11.129501428	7.617721448	13.981080161
14.967138870	-3.289257764	16.523286979
18.810157560	-3.259137292	16.639833296
16.828839034	0.015739488	16.765888100
22.610304277	-3.147452054	16.854963390
24.494504898	0.162854357	16.856326722
20.594586755	6.646407498	16.913295193
18.695822690	3.365206552	16.917464156
11.238911278	-10.093732516	16.922491298
20.726113153	-6.556176099	16.925920531
30.268762686	3.364902315	16.929115187
14.952596203	3.373264995	16.931102122
26.378691304	3.371766126	16.932193717
22.488617432	3.372654447	16.939674624
11.209350621	3.383879060	16.942830959
15.114232736	-10.080704594	16.956859112
13.078953869	6.690749776	16.973653413
18.841929827	-9.993234687	17.017723647
28.262888262	6.559419453	17.023685612
9.307950721	0.205220371	17.023723934
26.410433272	-2.975480068	17.025789835
7.415816961	-10.004534671	17.026023251
7.401261256	-2.873262483	17.089381034
22.569594345	-9.961258442	17.119346470
3.592730149	-9.932365695	17.142063253
5.578629077	1.430840124	0.817268476
9.502437408	1.514896530	0.879146147
13.127995366	8.358002731	0.992653418
5.531623380	8.387196919	1.076663680
20.787461480	8.458662179	1.129618217
16.904608411	14.816907005	1.172948141
9.300014816	14.868709566	1.181598593
24.547606508	14.861475503	1.233656920
3.647440563	4.683102495	1.364698802
16.876248504	1.877330558	1.372336045
20.800053711	1.911242485	1.471036385
1.763255575	1.103857413	1.560969420
9.347009143	3.753693205	3.978251060
13.270817302	3.837775626	4.040089901
16.896376025	10.680881975	4.153597172

9.300003698	10.710050300	4.237646482
24.555852920	10.781534705	4.290600729
9.367836700	-2.733179807	4.333930942
1.763243982	-2.681377104	4.342581250
17.010846517	-2.688566041	4.394600674
7.415821222	7.005981739	4.525642556
20.644628826	4.200183347	4.533318847
24.568445489	4.234115507	4.632019042
5.531614017	3.426736309	4.721913029
13.115389802	6.076572353	7.139194959
17.039197624	6.160628415	7.201072703
20.664755666	13.003734763	7.314579974
13.068383680	13.032929240	7.398589800
28.324233243	13.104413453	7.451544338
13.136217112	-0.410300709	7.494874261
5.531635484	-0.358478596	7.503525149
20.779227008	-0.365712763	7.555583185
11.184201204	9.328834720	7.686625067
24.413009486	6.523062495	7.694262746
28.336826148	6.556994847	7.792962651
9.300016984	5.749588906	7.882896121
16.883780899	8.399445471	10.300177471
20.807589401	8.483501917	10.362054634
13.127996610	-4.546306239	10.475523583
5.531623602	-4.517137914	10.559572892
20.787461109	-4.445672713	10.612526849
16.904620077	1.912552183	10.655857353
9.300015466	1.964374385	10.664507661
24.547607499	1.957166281	10.716526794
14.952592641	11.651733220	10.847569257
28.181389808	8.845915476	10.855245258
9.253001194	8.879847828	10.953945162
13.068396966	8.072468038	11.043839149
20.652161221	10.722298067	13.461160563
24.575969724	10.806379896	13.522999404
16.896375654	-2.223453495	13.636506675
9.300003668	-2.194259018	13.720516501
24.555841431	-2.122793966	13.773470458
20.672988362	4.235450476	13.816800962
13.068395448	4.287253132	13.825451270
28.315997914	4.280039014	13.877509886
7.415821364	-5.898353450	14.008551188
20.644630133	-8.704125416	14.016188867
1.716240563	-8.670193397	14.114888771
16.836788403	10.395340772	14.204822241
13.115389431	-6.827762924	16.622104172
17.039197421	-6.743706182	16.683981335
20.664755976	0.099399871	16.797488606
13.068383990	0.128593578	16.881499593
28.324233545	0.200079360	16.934453550
24.441392514	6.558304257	16.977784054
16.836787225	6.610125866	16.986434362
32.084378236	6.602917762	17.038453495
11.184201173	-3.575474406	17.169494796
24.413009114	-6.381272782	17.177171959
5.484620841	-6.347340430	17.275871863

3.523099390	-23.515437313	38.777817853
7.492595245	-23.033833282	38.926783371
13.073975322	18.787568699	0.047031706
5.530142378	6.046797140	0.136341297
13.180519564	6.037737816	0.182242991
11.419870594	2.819503014	0.183100245
16.943979656	18.916165238	0.228378490
11.231123640	16.037522581	0.234690942
7.510943085	9.505649993	0.238704421
18.781474632	9.523142112	0.238938218
15.133966904	9.525972472	0.242795833
9.284144039	12.598953853	0.249264149
20.727743135	19.065310477	0.253043832
11.097463197	9.490928662	0.254329703
26.404511174	9.520975619	0.259551122
14.925294968	15.919894026	0.259745944
22.817994540	9.501659693	0.268941867
9.343840130	6.086298503	0.293061701
24.618596704	12.592681743	0.305569697
14.958829158	2.931233196	0.335183703
18.924457458	15.922729412	0.336625458
16.903700826	12.517553045	0.337326829
20.803237733	6.124913371	0.345626542
30.221099886	16.121504953	0.348977607
16.966550204	6.216195366	0.425038824
22.527759405	15.989919967	0.428078160
24.616937149	6.262655465	0.430143335
18.886092727	3.129874936	0.444171017
26.526921314	16.062459368	0.453756620
13.065231660	12.702102606	0.454419016
28.399696551	12.757706156	0.465251497
20.780138623	12.649320534	0.516647391
24.492847228	19.331199529	0.614880137
28.276626497	19.310222163	0.764586692
20.841484368	-0.428078665	0.941491375
22.855589602	3.128204353	0.948271455
7.291479540	4.616093894	2.972972740
11.260975054	5.097697541	3.121938839
5.537204040	1.237508260	3.207975374
9.298533478	8.369669636	3.297323853
16.948910661	8.360610802	3.343225702
15.188250404	5.142356123	3.344083010
9.407208034	1.366104566	3.389322063
3.694352317	-1.512538308	3.395634732
11.279322730	11.828502346	3.399686987
22.549866073	11.846040428	3.399881790
18.902347226	11.848851160	3.403739533
13.052535646	14.921852702	3.410207812
13.190972195	1.515224003	3.414026361
14.865854637	11.813826978	3.415273276
30.172891834	11.843829060	3.420533834
7.388523733	-1.630193074	3.420728637
26.586375199	11.824538985	3.429885549
13.112220452	8.409151400	3.454044340
28.386975664	14.915560371	3.466513487
18.727221104	5.254106586	3.496166251

11.387686476	-1.627357125	3.497607969
20.672080467	14.840406002	3.498309377
24.571618055	8.447766207	3.506609271
22.684328861	-1.428581849	3.509960409
20.734930526	8.539048155	3.586021626
14.990998178	-1.560147025	3.589060672
28.385328926	8.585554253	3.591087090
22.654473218	5.452753956	3.605114662
18.990172041	-1.487627412	3.614739276
16.833634798	15.024955419	3.615401781
32.168075511	15.080559065	3.626234117
24.548518945	14.972173491	3.677629938
16.956074589	1.781112426	3.775862722
20.739855557	1.760161324	3.925530519
24.609863497	1.894800330	4.102435056
26.623969924	5.451057286	4.109254040
11.059859862	6.938946876	6.133955251
15.029366832	7.420570659	6.282921351
9.305595817	3.560381186	6.368958176
13.066914133	10.692549568	6.458267462
20.717290983	10.683489358	6.504169601
18.956631407	7.465234870	6.505026619
13.175599643	3.688977388	6.550304575
7.462732510	0.810315043	6.556617244
15.047714167	14.151401231	6.560630596
26.318247069	14.168894402	6.560864592
22.670739004	14.171724085	6.564722334
5.515764029	-2.628234545	6.571190614
16.959363123	3.838122591	6.574969970
18.634234278	14.136679767	6.576256078
11.089068241	14.166707616	6.581477733
11.156903501	0.692685896	6.581672246
30.354766976	14.147411911	6.590868351
16.880600093	10.732030148	6.614987949
20.850216010	-2.634506250	6.627495999
22.495601426	7.576985142	6.657110150
15.156078127	0.695541346	6.658551868
13.135309780	-2.709654441	6.659253276
28.340008471	10.770665092	6.667552880
3.600513916	0.894317080	6.670903727
24.503310848	10.861926903	6.746965234
18.759378711	0.762732093	6.750004281
9.301503972	10.908407234	6.752069601
26.422852178	7.775606937	6.766097174
22.758552321	0.835251261	6.775682885
9.296863176	-2.525105276	6.776345390
1.779111090	-2.469481493	6.787177726
17.011746474	-2.577887693	6.838573838
20.724466198	4.104011015	6.936806330
24.508236048	4.083014409	7.086513321
5.526051360	4.217673256	7.263417858
7.540156762	7.773956571	7.270197939
14.828251299	9.261845760	9.294898860
18.797747154	9.743449022	9.443865540
13.073975121	5.883259341	9.529901785
16.835294115	13.015402165	9.619250554

24.485671305	13.006342339	9.665152113
22.725010367	9.788087851	9.666009130
16.943979285	6.011855751	9.711248764
11.231124287	3.133188161	9.717599755
7.510943395	-3.398685320	9.721613688
18.781497585	-3.381166886	9.721808201
15.133966701	-3.378336610	9.725665943
9.284144179	-0.305355501	9.732134223
20.727743445	6.160975188	9.735953062
11.097463507	-3.413406399	9.737238589
3.552295600	-3.383378758	9.742459664
14.925295278	3.015559013	9.742654757
22.817995699	-3.402648992	9.751811379
20.648991870	13.054903265	9.775970460
24.618596332	-0.311627887	9.788440188
26.263981748	9.899837738	9.818093243
18.924458786	3.018394919	9.819534380
16.903690018	-0.386801608	9.820235787
32.108388793	13.093518073	9.828535391
7.368894919	3.217169676	9.831886819
28.271702626	13.184800021	9.907947746
22.527782189	3.085585074	9.910986792
13.069883102	13.231285982	9.913013210
30.191243955	10.098505437	9.927041363
26.526932980	3.158104450	9.936665977
13.065254785	-0.202232455	9.937327902
5.547491240	-0.146628216	9.948160237
20.780138252	-0.255014960	9.999556930
24.492846857	6.426864588	10.097788842
28.276627488	6.405912701	10.247456930
9.294429983	6.540551412	10.424361467
11.308536062	10.096809552	10.431180450
18.596630940	11.584698357	12.455881952
11.260975448	-7.806637204	12.604847471
16.842356117	8.206113122	12.690884877
9.298523006	-4.534684594	12.780233066
16.948910626	-4.543698308	12.826096302
15.188250756	-7.761953314	12.826953320
20.712359607	8.334708732	12.872231276
14.999503591	5.456065932	12.878543945
11.279323036	-1.075806572	12.882557297
22.549878076	-1.058313609	12.882790713
18.902347192	-1.055483333	12.886648455
13.052524501	2.017497480	12.893116734
24.496123767	8.483853936	12.896896671
14.865843829	-1.090527651	12.898182198
7.320686867	-1.060479873	12.903403272
18.693674919	5.338437761	12.903598366
26.586376190	-1.079796100	12.912794471
13.112221099	-4.495157134	12.936914069
28.386976655	2.011225478	12.949422119
18.727220732	-7.650203116	12.979036851
22.692837409	5.341273075	12.980477989
20.672080770	1.936071717	12.981218880
24.571617852	-4.456542622	12.989479000
11.137274901	5.540048424	12.992830428

20.734930323	-4.365286826	13.068930838
26.296150811	5.408483959	13.071930401
5.533123447	-4.318780728	13.073996302
22.654472931	-7.451580788	13.088023294
30.295324421	5.481002743	13.097609586
16.833634427	2.120646293	13.098271511
9.315871559	2.176251124	13.109103846
24.548518574	2.067863788	13.160500539
28.261227179	8.749743336	13.258732451
32.045008484	8.728767251	13.408438861
13.062810638	8.863405193	13.585344559
3.771764529	-7.453251588	13.592124059
11.059860000	-5.965362339	15.616825561
15.029367141	-5.483764618	15.765830563
9.305584882	-9.343948091	15.851828486
13.066914103	-2.211785709	15.941176675
20.717290949	-2.220844943	15.987078233
18.956631036	-5.439100022	15.987935251
13.175588238	-9.215351519	16.033174884
18.767884587	7.778920482	16.039525875
15.047703695	1.247046617	16.043540389
26.318246697	1.264559125	16.043773805
22.670727851	1.267396007	16.047592064
16.820915597	4.340396365	16.054060343
16.959362920	-9.066212774	16.057879763
18.634235943	1.232345674	16.059165290
11.089067189	1.262372723	16.064386365
22.462055923	7.661290358	16.064581458
7.502550384	1.243082648	16.073738080
16.880600740	-2.172304537	16.097897161
9.303150934	4.334104226	16.110365728
22.495601055	-5.327349751	16.140018782
26.461229187	7.664145808	16.141461081
24.440461092	4.258950465	16.142162488
5.487803844	-2.133670185	16.150462092
14.905666338	7.862921157	16.153813520
24.503310814	-2.042407782	16.229874447
30.064552927	7.731336555	16.232913493
9.301503597	-1.995901684	16.234939911
3.570648229	-5.128701596	16.248966903
11.211498700	7.803856108	16.258591517
20.602016104	4.443500074	16.259254603
13.084251881	4.499103720	16.270086938
28.316898896	4.390717153	16.321482469
20.724467441	-8.800323818	16.419715543
1.656031846	-8.821294954	16.569383631
5.526039021	-8.686656095	16.746288168
7.540156050	-5.130378707	16.753107151

CdS (100) surface: Structural parameters (VASP)

Cd S
1.0

8.3121795654	0.0000000000	0.0000000000
0.0000000000	13.5200080872	0.0000000000
12.4682638491	0.0000000000	36.5956770618

48 48
Cartesian

0.000000000	0.001230321	0.000000000
2.078036350	3.381232160	1.199752678
2.078048500	0.001230321	3.599294571
4.156084912	3.381232160	4.799047249
4.156084552	0.001230321	7.198552606
6.234120902	3.381232160	8.398305284
6.234133299	0.001230321	10.797847722
4.156079635	3.381232160	11.997599855
8.312177278	0.001230321	14.397105211
6.234136418	3.381232160	15.596894426
6.234136073	0.001230321	17.996399782
8.312172346	3.381232160	19.196151915
0.000000000	6.761234585	0.000000000
2.078036350	10.141236606	1.199752678
2.078048500	6.761234585	3.599294571
4.156084912	10.141236606	4.799047249
4.156084552	6.761234585	7.198552606
6.234120902	10.141236606	8.398305284
6.234133299	6.761234585	10.797847722
4.156079635	10.141236606	11.997599855
8.312177278	6.761234585	14.397105211
6.234136418	10.141236606	15.596894426
6.234136073	6.761234585	17.996399782
8.312172346	10.141236606	19.196151915
4.156089783	0.001230321	0.000000000
6.234126257	3.381232160	1.199752678
6.234138221	0.001230321	3.599294571
8.312174695	3.381232160	4.799047249
8.312174211	0.001230321	7.198552606
10.390210685	3.381232160	8.398305284
10.390231257	0.001230321	10.797847722
8.312177763	3.381232160	11.997599855
12.468267061	0.001230321	14.397105211
10.390226201	3.381232160	15.596894426
10.390225716	0.001230321	17.996399782
12.468262005	3.381232160	19.196151915
4.156089783	6.761234585	0.000000000
6.234126257	10.141236606	1.199752678
6.234138221	6.761234585	3.599294571
8.312174695	10.141236606	4.799047249
8.312174211	6.761234585	7.198552606
10.390210685	10.141236606	8.398305284
10.390231257	6.761234585	10.797847722
8.312177763	10.141236606	11.997599855
12.468267061	6.761234585	14.397105211
10.390226201	10.141236606	15.596894426

10.390225716	6.761234585	17.996399782
12.468262005	10.141236606	19.196151915
0.000000000	2.547291212	0.000000000
2.078036350	5.927293234	1.199752678
2.078048500	2.547291212	3.599294571
4.156084912	5.927293234	4.799047249
4.156084552	2.547291212	7.198552606
6.234120902	5.927293234	8.398305284
6.234133299	2.547291212	10.797847722
4.156079635	5.927293234	11.997599855
8.312177278	2.547291212	14.397105211
6.234136418	5.927293234	15.596894426
6.234136073	2.547291212	17.996399782
8.312172346	5.927293234	19.196151915
0.000000000	9.307294853	0.000000000
2.078036350	12.687296875	1.199752678
2.078048500	9.307294853	3.599294571
4.156084912	12.687296875	4.799047249
4.156084552	9.307294853	7.198552606
6.234120902	12.687296875	8.398305284
6.234133299	9.307294853	10.797847722
4.156079635	12.687296875	11.997599855
8.312177278	9.307294853	14.397105211
6.234136418	12.687296875	15.596894426
6.234136073	9.307294853	17.996399782
8.312172346	12.687296875	19.196151915
4.156089783	2.547291212	0.000000000
6.234126257	5.927293234	1.199752678
6.234138221	2.547291212	3.599294571
8.312174695	5.927293234	4.799047249
8.312174211	2.547291212	7.198552606
10.390210685	5.927293234	8.398305284
10.390231257	2.547291212	10.797847722
8.312177763	5.927293234	11.997599855
12.468267061	2.547291212	14.397105211
10.390226201	5.927293234	15.596894426
10.390225716	2.547291212	17.996399782
12.468262005	5.927293234	19.196151915
4.156089783	9.307294853	0.000000000
6.234126257	12.687296875	1.199752678
6.234138221	9.307294853	3.599294571
8.312174695	12.687296875	4.799047249
8.312174211	9.307294853	7.198552606
10.390210685	12.687296875	8.398305284
10.390231257	9.307294853	10.797847722
8.312177763	12.687296875	11.997599855
12.468267061	9.307294853	14.397105211
10.390226201	12.687296875	15.596894426
10.390225716	9.307294853	17.996399782
12.468262005	12.687296875	19.196151915

ZnS (110) surface: Structural parameters (VASP)

Zn S
1.0

10.7748718262	0.0000000000	0.0000000000
0.0000000000	15.2379703522	0.0000000000
0.0000000000	22.8569548267	42.8569553302

96 96
Cartesian

2.686337101	5.696303874	0.009771386
5.378923765	7.625232767	1.915277409
5.390593128	11.423921875	1.900105911
2.691627651	5.713728381	3.811911950
2.696777873	9.524089317	3.807111775
5.387985664	11.428104492	5.713817808
5.386153696	15.238396570	5.714889409
2.694181327	9.523975325	7.618681107
2.693491730	13.333078953	7.619152407
5.387274392	15.238091902	9.523843918
5.387511054	19.047485831	9.523629981
2.693750389	13.333277458	11.428536067
2.693750389	17.142777539	11.428407066
5.387274392	19.047592829	13.333099215
5.387511054	7.619007834	13.333355940
2.693491730	17.142564264	15.237790088
2.694181327	20.952429918	15.238261388
5.387985664	22.856588994	17.143124687
5.386153696	11.428904136	17.142096511
2.696777873	20.952574495	19.049831039
2.691627651	24.761198338	19.045031183
5.378923765	11.434717239	20.941665245
5.390593128	15.233413981	20.956879690
2.686337101	24.743773426	22.847170994
2.686337101	13.315288596	0.009771386
5.378923765	15.244217943	1.915277409
5.390593128	3.804936472	1.900105911
2.691627651	13.332713784	3.811911950
2.696777873	17.143074947	3.807111775
5.387985664	3.809119145	5.713817808
5.386153696	7.619411848	5.714889409
2.694181327	17.142960501	7.618681107
2.693491730	5.714094004	7.619152407
5.387274392	7.619106953	9.523843918
5.387511054	11.428500655	9.523629981
2.693750389	20.952262634	11.428536067
2.693750389	9.523792136	11.428407066
5.387274392	11.428607199	13.333099215
5.387511054	15.237992840	13.333355940
2.693491730	9.523578634	15.237790088
2.694181327	13.333444287	15.238261388
5.387985664	15.237604272	17.143124687
5.386153696	19.047889539	17.142096511
2.696777873	13.333589546	19.049831039
2.691627651	17.142213616	19.045031183
5.378923765	19.053702528	20.941665245
5.390593128	22.852398703	20.956879690

2.686337101	17.124773264	22.847170994
8.073773335	5.696303874	0.009771386
10.766359678	7.625232767	1.915277409
0.003157037	11.423921875	1.900105911
8.079063403	5.713728381	3.811911950
8.084214107	9.524089317	3.807111775
0.000549518	11.428104492	5.713817808
10.773589930	15.238396570	5.714889409
8.081616919	9.523975325	7.618681107
8.080927804	13.333078953	7.619152407
10.774709984	15.238091902	9.523843918
0.000075424	19.047485831	9.523629981
8.081185981	13.333277458	11.428536067
8.081185981	17.142777539	11.428407066
10.774709984	19.047592829	13.333099215
0.000075424	7.619007834	13.333355940
8.080927804	17.142564264	15.237790088
8.081616919	20.952429918	15.238261388
0.000549518	22.856588994	17.143124687
10.773589930	11.428904136	17.142096511
8.084214107	20.952574495	19.049831039
8.079063403	24.761198338	19.045031183
10.766359678	11.434717239	20.941665245
0.003157037	15.233413981	20.956879690
8.073773335	24.743773426	22.847170994
8.073773335	13.315288596	0.009771386
10.766359678	15.244217943	1.915277409
0.003157037	3.804936472	1.900105911
8.079063403	13.332713784	3.811911950
8.084214107	17.143074947	3.807111775
0.000549518	3.809119145	5.713817808
10.773589930	7.619411848	5.714889409
8.081616919	17.142960501	7.618681107
8.080927804	5.714094004	7.619152407
10.774709984	7.619106953	9.523843918
0.000075424	11.428500655	9.523629981
8.081185981	20.952262634	11.428536067
8.081185981	9.523792136	11.428407066
10.774709984	11.428607199	13.333099215
0.000075424	15.237992840	13.333355940
8.080927804	9.523578634	15.237790088
8.081616919	13.333444287	15.238261388
0.000549518	15.237604272	17.143124687
10.773589930	19.047889539	17.142096511
8.084214107	13.333589546	19.049831039
8.079063403	17.142213616	19.045031183
10.766359678	19.053702528	20.941665245
0.003157037	22.852398703	20.956879690
8.073773335	17.124773264	22.847170994
1.350824121	26.679079409	42.852668923
4.042882870	5.721057992	1.898434487
4.043378353	9.520272156	1.899420195
1.347570090	11.430161491	3.811569012
1.349110966	7.617407761	3.806254749
4.040458443	9.523556311	5.715789223
4.040910896	13.333376097	5.713603870

1.346934441	15.237886328	7.618466531
1.346848221	11.428576639	7.619409770
4.040587853	13.333269542	9.523587193
4.040555420	17.142663585	9.523972919
1.346858978	19.047454806	11.428621642
1.346858978	15.237954725	11.428321491
4.040587853	17.142739134	13.333355940
4.040555420	20.952147709	13.332970215
1.346848221	22.857054506	15.237576789
1.346934441	19.047363464	15.238519390
4.040458443	20.952041381	17.141153911
4.040910896	9.523891184	17.143339263
1.349110966	11.426900610	19.050731491
1.347570090	22.858646385	19.045373482
4.042882870	24.768520649	20.958551593
4.043378353	13.329772124	20.957522140
1.350824121	15.250602426	22.861271689
1.350824121	34.298064585	42.852668923
4.042882870	13.340042714	1.898434487
4.043378353	1.901287207	1.899420195
1.347570090	3.811176315	3.811569012
1.349110966	15.236392937	3.806254749
4.040458443	17.142541033	5.715789223
4.040910896	5.714391375	5.713603870
1.346934441	7.618901151	7.618466531
1.346848221	19.047562269	7.619409770
4.040587853	5.714284593	9.523587193
4.040555420	9.523677954	9.523972919
1.346858978	11.428470084	11.428621642
1.346858978	7.618969776	11.428321491
4.040587853	9.523754185	13.333355940
4.040555420	13.333162987	13.332970215
1.346848221	15.238069329	15.237576789
1.346934441	11.428378742	15.238519390
4.040458443	13.333056205	17.141153911
4.040910896	17.142875991	17.143339263
1.349110966	19.045870686	19.050731491
1.347570090	15.239661209	19.045373482
4.042882870	17.149535473	20.958551593
4.043378353	20.948757073	20.957522140
1.350824121	22.869587148	22.861271689
6.738260034	26.679079409	42.852668923
9.430318462	5.721057992	1.898434487
9.430814266	9.520272156	1.899420195
6.735005843	11.430161491	3.811569012
6.736546558	7.617407761	3.806254749
9.427894035	9.523556311	5.715789223
9.428346809	13.333376097	5.713603870
6.734370033	15.237886328	7.618466531
6.734283973	11.428576639	7.619409770
9.428023766	13.333269542	9.523587193
9.427991012	17.142663585	9.523972919
6.734294891	19.047454806	11.428621642
6.734294891	15.237954725	11.428321491
9.428023766	17.142739134	13.333355940
9.427991012	20.952147709	13.332970215

6.734283973	22.857054506	15.237576789
6.734370033	19.047363464	15.238519390
9.427894035	20.952041381	17.141153911
9.428346809	9.523891184	17.143339263
6.736546558	11.426900610	19.050731491
6.735005843	22.858646385	19.045373482
9.430318462	24.768520649	20.958551593
9.430814266	13.329772124	20.957522140
6.738260034	15.250602426	22.861271689
6.738260034	34.298064585	42.852668923
9.430318462	13.340042714	1.898434487
9.430814266	1.901287207	1.899420195
6.735005843	3.811176315	3.811569012
6.736546558	15.236392937	3.806254749
9.427894035	17.142541033	5.715789223
9.428346809	5.714391375	5.713603870
6.734370033	7.618901151	7.618466531
6.734283973	19.047562269	7.619409770
9.428023766	5.714284593	9.523587193
9.427991012	9.523677954	9.523972919
6.734294891	11.428470084	11.428621642
6.734294891	7.618969776	11.428321491
9.428023766	9.523754185	13.333355940
9.427991012	13.333162987	13.332970215
6.734283973	15.238069329	15.237576789
6.734370033	11.428378742	15.238519390
9.427894035	13.333056205	17.141153911
9.428346809	17.142875991	17.143339263
6.736546558	19.045870686	19.050731491
6.735005843	15.239661209	19.045373482
9.430318462	17.149535473	20.958551593
9.430814266	20.948757073	20.957522140
6.738260034	22.869587148	22.861271689
